# Expression dynamics of integrin α2, α3, and αV upon osteogenic differentiation of human mesenchymal stem cells

**DOI:** 10.1186/s13287-020-01714-7

**Published:** 2020-06-03

**Authors:** Hyun Min Lee, Se-Ri Seo, Jeeseung Kim, Min Kyu Kim, Hyosun Seo, Kyoung Soo Kim, Young-Joo Jang, Chun Jeih Ryu

**Affiliations:** 1 Department of Integrative Bioscience and Biotechnology, Institute of Anticancer Medicine Development, Sejong University, 209 Neungdong-ro, Gwangjin-gu, Seoul, 05006 Korea; 2 Department of Clinical Pharmacology and Therapeutics, Kyung Hee University School of Medicine, Seoul, 02447 Korea; 3 Department of Nanobiomedical Science, BK21 PLUS NBM Global Research Center for Regenerative Medicine, College of Dentistry, Dankook University, Cheonan, 330-714 Korea

**Keywords:** Human mesenchymal stem cells, Osteoblasts, Monoclonal antibodies, Osteogenic differentiation, Integrin αV, Integrin α3, Integrin α2

## Abstract

**Background:**

The differentiation of human mesenchymal stem cells (hMSCs) into osteoblasts (OBs) is a prerequisite for bone formation. However, little is known about the definitive surface markers for OBs during osteogenesis.

**Methods:**

To study the surface markers on OBs, we generated and used monoclonal antibodies (MAbs) against surface molecules on transforming growth factor-β1 (TGF-β1)-treated cancer cells. The generated MAbs were further selected toward expression changes on hMSCs cultured with TGF-β1/bone morphogenetic protein-2 (BMP-2) or osteogenic differentiation medium (ODM) by flow cytometry. Immunoprecipitation and mass spectrometry were performed to identify target antigens of selected MAbs. Expression changes of the target antigens were evaluated in hMSCs, human periodontal ligament cells (hPDLCs), and human dental pulp cells (hDPCs) during osteogenic and adipogenic differentiation by quantitative polymerase chain reaction (qPCR) and flow cytometry. hMSCs were also sorted by the MAbs using magnetic-activated cell sorting system, and osteogenic potential of sorted cells was evaluated via Alizarin Red S (ARS) staining and qPCR.

**Results:**

The binding reactivity of MR14-E5, one of the MAbs, was downregulated in hMSCs with ODM while the binding reactivity of ER7-A7, ER7-A8, and MR1-B1 MAbs was upregulated. Mass spectrometry and overexpression identified that MR14-E5, ER7-A7/ER7-A8, and MR1-B1 recognized integrin α2, α3, and αV, respectively. Upon osteogenic differentiation of hMSCs, the expression of integrin α2 was drastically downregulated, but the expression of integrin α3 and αV was upregulated in accordance with upregulation of osteogenic markers. Expression of integrin α3 and αV was also upregulated in hPDLCs and hDPCs during osteogenic differentiation. Cell sorting showed that integrin αV-high hMSCs have a greater osteogenic potential than integrin αV-low hMSCs upon the osteogenic differentiation of hMSCs. Cell sorting further revealed that the surface expression of integrin αV is more dramatically induced even in integrin αV-low hMSCs.

**Conclusion:**

These findings suggest that integrin α3 and αV induction is a good indicator of OB differentiation. These findings also shed insight into the expression dynamics of integrins upon osteogenic differentiation of hMSCs and provide the reason why different integrin ligands are required for OB differentiation of hMSCs.

## Background

Human mesenchymal stem cells (hMSCs) can differentiate into multilineage cells of mesenchymal tissues, including osteoblasts (OBs), adipocytes, myocytes, and chondrocytes [1]. Although hMSCs can differentiate into a variety of cell types, the differentiation of hMSCs into osteocytes and adipocytes has been extensively studied for successful bone formation and regeneration [2–4]. Osteocytes compose approximately 95% of all bone cells in adult bones and are differentiated from hMSCs through OBs [5]. Although many researchers have tried to differentiate hMSCs into OBs and osteocytes for bone formation and regeneration, they encountered numerous problems because hMSCs are heterogeneous populations with variable functions, and their properties are highly variable depending on the tissue source, subpopulation, and culture condition [6, 7]. Furthermore, there are no definitive markers to identify and isolate OBs from the mixed populations.

Transforming growth factor-β1 (TGF-β1), bone morphogenetic protein 2 (BMP-2), and runt-related transcription factor 2 (Runx2) are believed to be key regulators in OB development during osteogenesis. TGF-β1 is the predominant TGF-β isoform expressed in bone and has been extensively studied during bone formation and remodeling [8, 9]. TGF-β1 signaling is known to expand MSCs and stimulate early differentiation of OBs, whereas inhibiting late differentiation of OBs into osteocytes [10]. BMP signaling affects OB commitment, and treatment of MSCs with BMP-2 promotes osteogenesis [2]. Treatment of MSCs with both TGF-β1 and BMP-2 accelerates bone formation through the proliferation of OBs and keeps immature OBs. Runx2 is a key transcription factor for early OB differentiation and bone formation and is controlled by TGF-β1 and BMP-2 during osteogenesis [2, 11–13]. Runx2 is also upregulated in TGF-β1-treated cancer cells [14].

Epithelial-mesenchymal transition (EMT) is regarded to play important roles in cancer metastasis and recurrence [15, 16] and is induced by cytokines, such as TGF-β1 [17]. BMP-2, BMP-4, and BMP-7 also promote EMT and invasiveness in pancreatic cancer cells [17–19]. Runx2 also takes part in TGF-β-induced EMT [14]. Cancer cells undergoing EMT show enhanced ability of invasion and metastasis and also reveal similarity to MSC-like properties [17]. To search for novel cell surface markers on EMT-phenotypic-circulating tumor cells, in the previous study, we generated a panel of monoclonal antibodies (MAbs) against surface molecules on TGF-β1-treated A549 human non-small cell lung carcinoma cells (NSCLC) because TGF-β1-treated A549 cells represent EMT-phenotypic cells [20–22]. Although EMT plays major roles in tumor invasion and metastasis, it also occurs during the process of odontogenesis. EMT has an important role in differentiation of enamel-forming ameloblast [23]. Studies have shown that epithelial cells differentiate into hard tissues such as cementum in the presence of TGF-β-1 during EMT [23–26]. A recent study also suggested that EMT drives odontogenic epithelial stem cells to develop supernumerary teeth through interaction with various MSCs [27].

In this study, therefore, we postulated that the surface molecules expressed on EMT-phenotypic cells may be source molecules for finding novel surface markers on TGF-β1-regulated OBs. To this end, we first generated a panel of MAbs against TGF-β1-treated A549 cells by using the decoy immunization strategy [28]. To use the MAbs for the identification and isolation of novel surface molecules on OBs, the MAbs showing hMSC-binding reactivity were then selected for their binding capacity for differentiated hMSCs treated with TGF-β1/BMP-2 or osteogenic differentiation medium (ODM). To examine whether the cell surface expression of the MAb antigens is controlled by Runx2 expression, the cell surface expression of the MAb antigens was also examined in Runx2 knockdown osteosarcoma cells. Integrin α2, α3, and αV recognized with the MAbs were identified by immunoprecipitation followed by mass spectrometry. Upon osteogenic differentiation of hMSCs, integrin α2 expression was rapidly downregulated, while integrin α3 and αV expression was gradually upregulated in accordance with upregulation of osteogenic markers. Integrin α3 and αV expression was also upregulated in human periodontal ligament cell (hPDLCs) and human dental pulp cells (hDPCs). To further elucidate the role of integrin αV during osteogenic differentiation of hMSCs, integrin αV-high and αV-low hMSCs were sorted and subjected to osteogenic differentiation. Based on the results, we further discuss and propose the biological roles of integrin αV in osteogenic differentiation of hMSCs.

## Methods

### Cell culture and differentiation

Human non-small cell lung carcinoma cell line (NSCLC) A549 and human osteosarcoma cell lines (U2OS and SAOS-2) were purchased from the Korean Cell Line Bank (KCLB, Seoul, Korea) and were cultured according to the protocol provided by the supplier. Human fetal osteoblast cell line hFOB1.19 was purchased from ATCC (CRL-11372, Manassas, VA) and was cultured according to the protocol provided by the supplier. Human bone marrow-derived mesenchymal stem cells (hMSCs) were kindly provided by Prof. Sung-Soo Kim (Ajou University School of Medicine, Suwon, Korea) [29]. hMSCs were cultured in DMEM (Welgene, Daegu, Korea) supplemented with 20 ng/ml basic fibroblast growth factor (bFGF) (R&D system, Minneapolis, MN), 10% fetal bovine serum (FBS) (Welgene), and antibiotic-antimycotic solution (Welgene). The medium was changed every 2 days, and early passages (passages 4–6) were used in this study. Human peripheral blood mononuclear cells (PBMCs) were isolated by the Ficoll-Paque Plus method (GE Healthcare, Seoul, Korea). For the primary cultures of hPDLCs and hDPCs, adult third molars were collected from patients who were 19 to 23 years old under guidelines approved by the Institutional Review Board of the Dankook Dental Hospital and Dankook University (DKU NON2019-004), and the informed consent for all experiments using extracted teeth was obtained from all the participants. Human periodontal ligament and pulp tissues were separated from the surface of the tooth root and pulp part inside of the tooth after removing tooth crown. Tissues were enzymatically digested with 3 mg/ml collagenase type I (Merck Millipore, Seoul, Korea) and 4 mg/ml dispase (Sigma-Aldrich, Gyeonggi, Korea) at 37 °C for 1 h. Cell suspensions were incubated in α-MEM (GE Healthcare, Seoul, Korea) containing 20% FBS (GE Healthcare) and 1% antibiotics (Lonza, Seoul, Korea) at 37 °C in humidified atmosphere supplemented with 5% CO_2_ [30, 31]. To induce EMT in A549 cells, the cells were plated at 1.8 × 10^4^ cells/cm^2^ and incubated for 24 h. The cells were then treated with 5 ng/ml of TGF-β1 (Peprotech, Rocky Hill, NJ) for 4 days and the medium was changed every 2 days.

### Generation of a panel of murine MAbs

To generate hybridomas producing antibodies specific to TGF-β1-treated A549 cells, one million TGF-β1-treated A549 cells were injected into the left hind footpads of female BALB/c mice (DBL, Chungbuk, Korea) 3 days after the decoy cells (A549 cells) were injected into the right hind footpad of the same mice. The following procedures were carried out as described previously [28]. Briefly, lymphocyte fractions isolated from the left popliteal lymph nodes were fused to FO myeloma cells (ATCC) by polyethylene glycol 1500 (Roche, Seoul, Korea). The fused cells were maintained in DMEM (Corning, Seoul, Korea) supplemented with 20% FBS (Welgene), HAT supplement (Sigma-Aldrich), and antibiotic-antimycotic solution (Welgene). Hybridoma clones specific to TGF-β1-treated A549 cells were selected by flow cytometry. Isotype of antibodies secreted from hybridomas was analyzed with the Mouse Immunoglobulin Isotyping Kit (BD Biosciences, Seoul, Korea) according to the manufacturer’s instructions. Antibody purification was performed by using Protein G agarose column chromatography (Merck Millipore). All animal experiments were approved by the Institutional Animal Care and Use Committee at Sejong University (SJ-21051104).

### Flow cytometry

To screen MAbs specific to OBs, cells (A549, TGF-β1-treated A549, SAOS-2, U2OS, hMSCs, and hFOB) were dissociated by trypsin-EDTA (Welgene) and filtered through a 40-μm strainer and washed by phosphate-buffered saline (PBS, pH 7.4). Cells were resuspended in ice-cold PBA (1% bovine serum albumin and 0.01% NaN_3_ in PBS) and incubated with primary antibodies, followed by the incubation with fluorescein isothiocyanate (FITC)-conjugated anti-mouse IgG (Santa Cruz Biotechnology, Santa Cruz, CA), Alexa488-conjugated anti-mouse IgG (Thermo Fisher Scientific, Seoul, Korea), phycoerythrin (PE)-conjugated anti-mouse IgG (Thermo Fisher Scientific), and/or DyLight 650-conjugated anti-mouse IgG (Thermo Fisher Scientific). To analyze only live cells, propidium iodide (PI) (Sigma-Aldrich)-negative cells were gated and analyzed by FACSCalibur (BD biosciences) and Cell Quest Software (BD biosciences). For multicolor flow cytometry, cells were incubated with appropriate primary antibodies for 30 min and incubated with Dylight 649-(Vector Laboratories, Burlingame, CA) or Alexa 488-conjugated anti-mouse IgG (Thermo Fisher Scientific) after osteogenic or adipogenic differentiation of hMSCs. The cells were than incubated with PE-conjugated mouse monoclonal anti-human CD73, CD90, or CD146 antibodies, rabbit polyclonal anti-human CD164, or rabbit polyclonal anti-integrin α2, α3, or αV antibodies.

### Cell surface biotinylation, immunoprecipitation, and Western blotting

A549 cells were incubated with 0.5 mg/ml of EZ-Link Sulfo NHS-LC-biotin (Thermo Fisher Scientific) for 30 min at 4 °C on a rocker. After three washes with ice-cold PBS (pH 8.0), the cells were lysed in ice-cold lysis buffer (25 mM Tris-HCl, pH 7.5, 250 mM NaCl, 5 mM EDTA, 1% Nonidet P-40, 2 μg/ml aprotinin, 100 μg/ml PMSF, 5 μg/ml leupeptin, 1 mM NaF, and 1 mM NaVO_3_) for 30 min at 4 °C, centrifuged at 12,000 rpm for 40 min at 4 °C, and stored at − 70 °C before analysis. To eliminate nonspecific proteins bound to Protein G agarose (Merck Millipore), biotin-labeled or non-labeled A549 lysates were incubated with 20 μl of Protein G agarose beads for 2 h at 4 °C (Preclearing), and the bead-bound proteins were used for negative control of immunoprecipitation (IP). Pre-cleared lysates were then incubated with MAbs, rabbit polyclonal anti-integrin α2 (Mybiosource, Seoul, Korea), anti-integrin α3 (Sino Biological, Wayne, PA), anti-integrin αV (Sino Biological), anti-His (Thermo Fisher Scientific), and/or anti-FLAG antibodies (MBL, Woburn, MA) at 4 °C overnight and further incubated with 20 μl of Protein G agarose at 4 °C for 3 h. The beads were washed by lysis buffer and bound proteins were eluted by acidic solution (0.1 M Glycine-HCl, pH 2.8) for 10 min at RT and neutralized by alkaline solution (1.0 M Tris-HCl, pH 9.0). Eluted proteins were denatured by boiling in sample buffer for 10 min at 100 °C before further analysis. For Western blotting, prepared proteins were denatured by sodium dodecyl sulfate (SDS)-polyacrylamide gel electrophoresis (SDS-PAGE) on 10–12% polyacrylamide gel and transferred to nitrocellulose membranes (Pall Corporation, New York, NY) for 2 h. The membranes were blocked by 5% skim milk for 30 min at RT and washed by 0.1% Tris-buffered saline with Tween-20 (TBST; 0.1% tween-20) for three times. Then, the membranes were incubated with streptavidin-conjugated horse radish peroxidase (SA-HRP) (1:5000, GE Healthcare) or HRP-conjugated anti-mouse (Merck Millipore) or rabbit IgG (Abcam, Hanam, Korea) for 1 h at RT and analyzed with West-Queen™ Western blot detection system (iNtRON Biotechnology, Seongnam, Korea).

### DNA and siRNA transfection

HEK293T cells (7 × 10^5^ cells) were transfected with His- or FLAG-tagged ITGA2 (Sino Biological), ITGA3 (Sino Biological), and ITGAV (Sino Biological) expression vectors using Lipofectamine™ 2000 (Thermo Fisher Scientific) according to the supplier’s protocol. Protein was transiently expressed for 24 h, and the cell pellets were lysed in RIPA buffer (50 mM Tris-HCl, pH 7.4, 150 mM NaCl, 1% NP-40, 0.5% sodium deoxycholic acid, 0.1% SDS). Cell lysates were subjected to IP and Western blot analysis as described above. siRNA oligonucleotides targeting RUNX2 (Santa Cruz Biotechnology) or control siRNA (Bioneer, Daejeon, Korea) were used in this study. U2OS cells (2.6 × 10^4^ cells/cm^2^) were transfected with 50 nM control or RUNX2 siRNA using Lipofectamine®RNAiMAX Reagent (Thermo Fisher Scientific) according to the supplier’s protocol. After 24 h, the cells were transfected again (double knockdown) and then harvested for analysis after 48 h.

### Antibody biotinylation and cell sorting

Antibody was biotinylated by using DSB-X biotin labeling kit (Thermo Fisher Scientific) according to the manufacturer’s procedure. A total of 5 million hMSC cells were incubated with 20 μg of biotinylated MR1-B1antibody for 30 min at RT with rolling and tilting. Cell sorting was carried out by using Dynabeads FlowComp Flexi kit (Thermo Fisher Scientific) according to the manufacturer’s protocol. Bead-unbound and bead-bound hMSCs were isolated and further analyzed with Alexa488-conjugated anti-mouse IgG (Thermo Fisher Scientific) and FACSCalibur.

### Osteogenic and adipogenic differentiation

To induce OB differentiation, hMSCs were also incubated for 14 days in 1 ng/ml of TGF-β1 and 10 ng/ml of BMP-2. To induce osteogenic and osteoblastic differentiation of hMSCs, hMSCs (500 cells/cm^2^) were incubated for 0, 4, 7, 12, 14, or 21 days in osteogenic differentiation medium (ODM) (60 μM L-ascorbic acid (Sigma-Aldrich), 10 mM β-glycerophosphate (Sigma-Aldrich), and 100 nM dexamethasone (Sigma-Aldrich) in DMEM containing 10% FBS and 1% antibiotics-antimycotics). To promote osteogenic differentiation of hMSCs, hMSCs were also incubated with ODM containing 1 μM SB431542 (MedChemExpress, Monmouth Junction, NJ), a specific inhibitor of TGF-β type I receptor, after 7 days of ODM incubation. For adipogenic differentiation of hMSCs, hMSCs (500 cells/cm^2^) were incubated for 0, 4, 7, 14, or 21 days in adipogenic differentiation medium (ADM) (60 μM indomethacin (Sigma-Aldrich), 500 mM 3-isobutyl-1-methylxanthine (Sigma-Aldrich), 10 μg/ml insulin (Sigma-Aldrich), and 1 μM dexamethasone (Sigma-Aldrich) in DMEM containing 10% FBS and 1% antibiotics-antimycotics). The differentiated cells were stained with Alizarin Red S (Sigma-Aldrich) or Oil Red O (Sigma-Aldrich). For osteoblastic differentiation of hPDLCs, cells were treated with 100 ng/ml BMP-2 (Prospec, Ness-Ziona, Israel) for 7 days. For odontoblastic differentiation of hDPCs, cells were treated with 100 ng/ml BMP-2 (Prospec) and 10 ng/ml BMP-4 (Prospec) for 7 days [31]. To induce osteoblastic and odontoblastic maturation, cells were cultured in α-MEM supplemented with 5% FBS, 100 nM dexamethasone, 100 μM ascorbic acid, and 5 mM β-glycerophosphate for 7 days, followed to treatment with cytokines for 7 days. For flow cytometric analysis, cells were detached by TrypLE Express (Thermo Fisher Scientific) and analyzed in FACSCalibur.

### Reverse transcription-polymerase chain reaction (RT-PCR)

U2OS cells were transfected with control or RUNX2 siRNAs as described above. The cells were collected by Trypsin-EDTA (Welgene), and total RNA was extracted by RNAiso Plus (TaKaRa, Otsu, Japan) according to the manufacturer’s instructions. To perform RT-PCR, cDNAs were synthesized by Prime Script™ RT Master Mix (TaKaRa) and PCR was conducted by e-Taq DNA Polymerase (SolGent, Daejeon, Korea). PCRs consisted of denaturation at 95 °C for 1 min, annealing at 56–67 °C for 45 s, and extension at 72 °C for 45 s in 30 cycles. Each gene was analyzed in triplicate. Primer sequences used are listed in Additional file 1 (Table S1).

### Quantitative PCR

Total RNAs were extracted from hMSCs and differentiated hMSCs using the RNAiso Plus (TaKaRa) according to the protocol provided by the manufacturer. cDNAs were generated from total RNAs by RT in a reaction containing 500 ng total RNAs and Prime Script RT Master Mix (TaKaRa). The RT reaction was performed at 37 °C for 15 min followed by heating at 94 °C for 5 s. Quantitative real-time PCR was performed using Applied StepOne™ Real-Time PCR System with SYBR Green reagents (Thermo Fisher Scientific). The standard reaction contained 12.5 μl 2 × PCR buffer (PowerUP™SYBR™ Green Master Mix, Thermo Fisher Scientific), 2 μl cDNA template, and 10 pM of forward and reverse primers in a total volume of 25 μl. The PCR was carried out for 30 cycles consisting of 1 min at 95 °C, 45 s at 45–56 °C, and 1 min at 72 °C. The target gene-specific primers for human transcripts encoding ITGA2, ITGA3, ITGAV, RUNX2, ALP, COL1A1, OSX2, C/EBPA, and GAPDH are listed in Additional file 1 (Table S1). Each sample was analyzed in triplicate for each target gene. To analyze gene expression in hPDLCs and hDPCs, total RNAs were extracted from hPDLCs or hDPCs using Easy-spin™ Total RNA Extraction kit (iNtRON Biotechnology). cDNAs were synthesized from total RNAs by using the ReverTra Ace™ qPCR RT kit (Toyobo, Osaka, Japan), and the qRT-PCR was performed using StepOn™ system (Thermo Fisher Scientific) with iTaq™ Universal SYBR™ Green Supermix (Bio-Rad, Hercules, CA) system. During qRT-PCR, a dissociation curve was constructed in the range of 65 to 95 °C, and the cycling parameters were followed as follows: 1 cycle for 30 s at 95 °C, 40 cycles for 15 s at 95 °C and 1 min at 55–60 °C for amplification. The threshold cycle was obtained, and the relative comparison of each target gene was analyzed. The primer sequences used are listed in Additional file 1 (Table S1).

### Statistical analysis

The paired samples *t*-test was a statistical procedure used to compare the difference between two populations, and a *p* value of less than 0.05 was considered statistically significant.

## Results

### Generation of a panel of MAbs against TGF-β1-treated A549 cells

In this study, we postulated that surface molecules expressed on TGF-β1-treated A549 cells may be source molecules for finding novel surface markers on TGF-β1-regulated OB cells. To this end, we first generated a panel of MAbs against TGF-β1-treated A549 cells by using the modified decoy immunization strategy [22, 28]. TGF-β1-treated A549 cells showed fibroblast-like morphologies and enhanced expression of the mesenchymal markers, including ZEB1, vimentin, slug, and hnRNPA2/B1, concomitant with downregulation of the epithelial marker E-cadherin (Additional file 1: Figure S1a, 1b). Flow cytometric analysis also showed that the cell surface expression of epithelial markers E-cadherin and EpCAM were downregulated in the TGF-β1-treated A549 cells, while the cell surface expression of mesenchymal marker N-cadherin was slightly upregulated (Additional file 1: Figure S1c). The results indicate that TGF-β1 induces A549 epithelial cells to undergo the EMT process, consistent with previous studies [20, 32]. To generate MAbs specific to TGF-β1-treated A549 cells, A549 cells were used as decoy immunogen by injection into right foot pads of BALB/c mice. TGF-β1-treated A549 cells were then used for target immunogen by injection into left foot pads of the same BALB/c mice 3 days after the first injection. Hybridomas were generated by fusion of FO myeloma cells and lymphocytes isolated from left popliteal regions in the immunized mice. From 263 hybridomas, 13 MAbs were selected and further analyzed because they showed increased binding affinity for TGF-β1-treated A549 cells as compared with parental A549 cells (Additional file 1: Figure S2 and Table S2). The 13 MAbs did not bind to PBMCs and were purified prior to further analysis.

### Binding reactivity of EMT-specific MAbs to mesenchymal stem/progenitor cells with osteogenic potential

Next, we examined the binding reactivity of the 13 EMT-specific MAbs to mesenchymal stem/progenitor cells, including hMSCs and hFOB, because they have strong osteogenic potential [1, 33, 34]. Two human osteosarcoma cell lines (U2OS and SAOS-2) were also included in the analysis because they have been frequently used as osteoblastic cancer cell lines [35, 36]. ER4-D2, ER7-C3, MR11-B3, and MR17-B8 did not bind to both hMSCs and hFOB while MR1-G2 bound to only hMSCs (Additional file 1: Figure S3). The rest of them (9 MAbs) were able to bind to hMSCs, hFOB, and one of the osteosarcoma cell lines (Additional file 1: Figure S3 and Table S3).

### Expression changes of target antigens of selected MAbs upon osteogenic differentiation of hMSCs

**Fig. 1:**
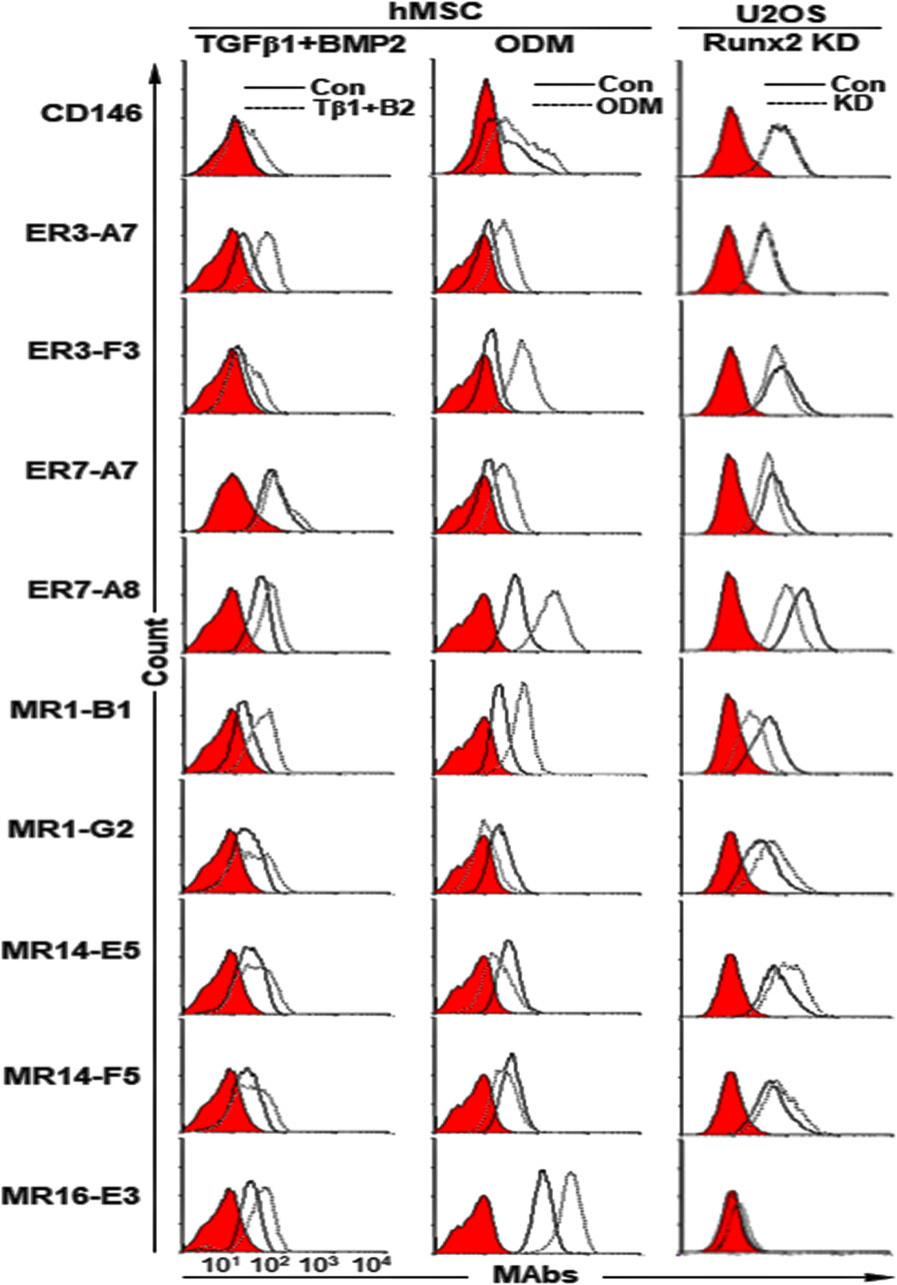
Expression changes of target antigens of selected MAbs upon osteogenic differentiation of hMSCs. hMSCs were treated for 14 days with TGF-β1 and BMP-2 and incubated for 12 days with ODM. Expression changes of target antigens of the selected MAbs were analyzed by flow cytometry after detachment of the cells. Runx2 knockdown U2OS cells were also analyzed by flow cytometry with the same MAbs. Red-filled histograms represent the isotype controls

Next, we examined the cell surface expression of target antigens of selected MAbs upon osteogenic differentiation of hMSCs. To promote the differentiation of hMSCs into OBs, hMSCs were treated with TGF-β1 and BMP-2 for 14 days, and the differentiated hMSCs were subjected to flow cytometric analysis. CD146, a marker of self-renewing osteoprogenitors in human bone marrow [37], was increased during differentiation, and all target antigens of the selected MAbs were also increased (Fig. 1, the first column). hMSCs were also cultured for 12 days in ODM [38]. Calcium deposition was readily detected by Alizarin Red S staining (Additional file 1: Figure S4A). After 12 days of osteogenic differentiation, CD146 was slightly increased in the ODM-differentiated hMSCs, and target antigens of 6 MAbs (ER3-A7, ER3-F3, ER7-A7, ER7-A8, MR1-B1 and MR16-E3) were also increased, while those of 3 MAbs (MR1-G2, MR14-E5 and MR14-F5) were decreased (Fig. 1, Table 1). Runx2 is a key transcription factor positively associated with OB phenotype and inhibits MSCs from adipogenic or chondrogenic differentiation [39]. Runx2 also keeps the immature state of OB preventing the maturation of OB into osteocyte [39]. Cell surface expression of target antigens of 4 MAbs (ER3-F3, ER7-A7, ER7-A8, and MR1-B1) was downregulated in Runx2 knockdown U2OS cells while cell surface expression of target antigens of 3 MAbs (MR1-G2, MR14-E5, and MR14-F5) was upregulated (Fig. 1, Table 1, Additional file 1: Figure S4B). Taken together, the results suggest that target antigens of 4 MAbs (ER3-F3, ER7-A7, ER7-A8 and MR1-B1) are upregulated upon osteogenic differentiation of hMSCs, while target antigens of 3 MAbs (MR1-G2, MR14-E5, and MR14-F5) are downregulated.

**Table 1:** Expression changes of target antigens of selected MAbs in hMSCs stimulated with osteogenic inducers and in Runx2 knockdown U2OS cells

Clone	Isotype	hMSCs		Runx2 knockdown U2OS
		TGF-β1 + BMP-2	ODM	
CD146	–	↑	↑	─
ER3-A7	IgG1, κ	↑↑	↑↑	─
ER3-F3	IgG2a, κ	↑	↑↑	↓
ER7-A7	IgG2a, κ	↑	↑↑	↓
ER7-A8	IgG1, κ	↑	↑↑↑	↓↓
MR1-B1	IgG2a, κ	↑↑	↑↑↑	↓↓
MR1-G2	IgG1, κ	↑	↓↓	↑
MR14-E5	IgG2a, κ	↑	↓↓	↑
MR14-F5	IgG2a, κ	↑	↓	↑
MR16-E3	IgG2a, κ	↑	↑↑↑	─

↑ weak upregulation; ↑↑ medium upregulation; ↑↑↑, strong upregulation; ↓ weak downregulation; ↓↓ medium downregulation; ─ no change

### Identification of target antigens of MR14-E5, ER7-A7, ER7-A8, and MR1-B1

**Fig. 2:**
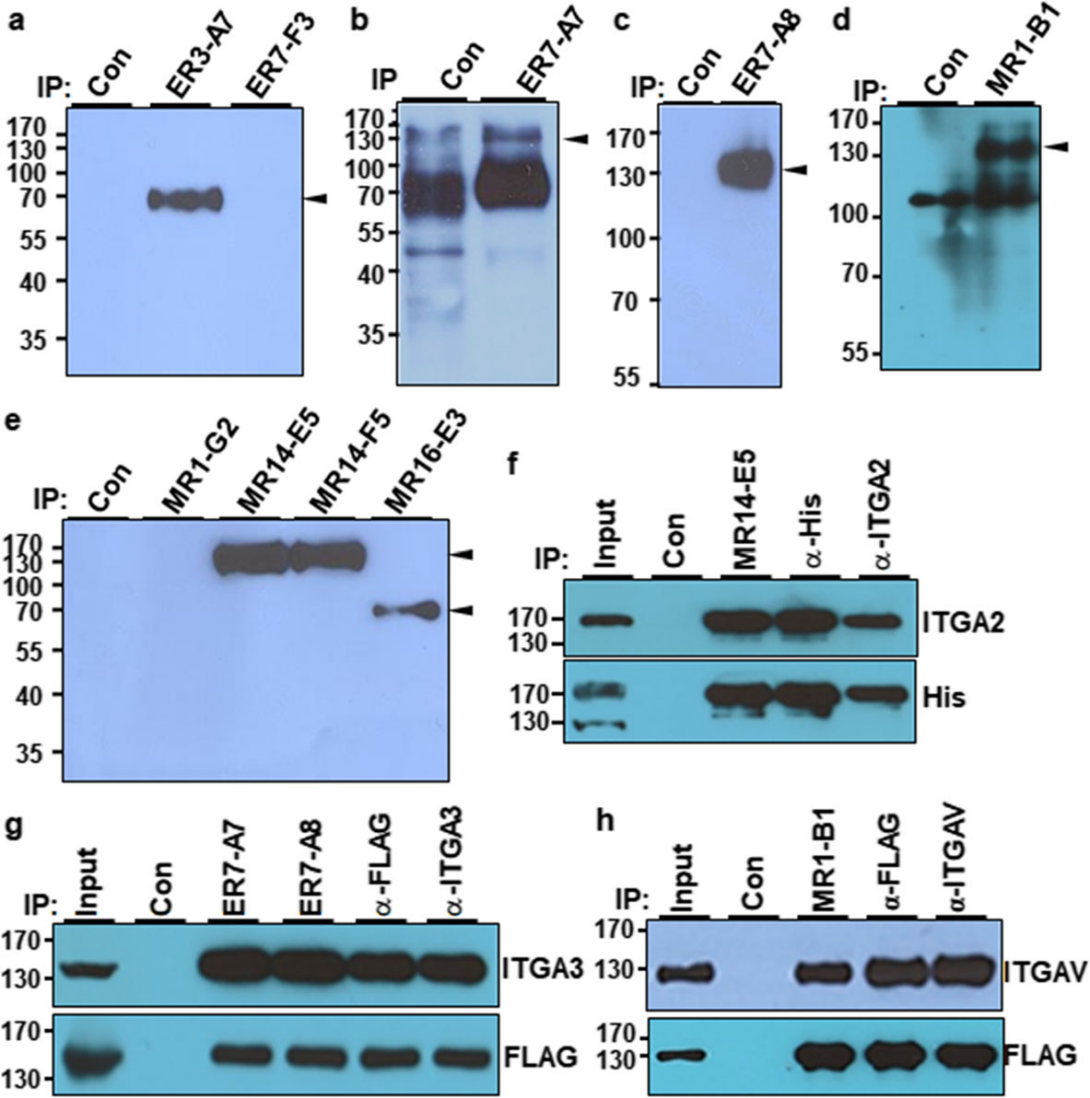
Identification of cell-surface molecules recognized by selected MAbs. **a–e** A549 cell lysates were immunoprecipitated with the indicated MAbs after cell surface biotinylation. After bead-bound proteins were eluted, the proteins were detected with SA-HRP in Western blotting. Preclearing was done with protein G agarose beads and used as a negative control. **f** Overexpression and immunoprecipitation of integrin α2 (ITGA2) with MR14-E5, anti-His, or anti-ITGA2 antibodies. HEK293T cells were transfected with pcDNA3.1+ (control vector) or pCMV-His-ITGA2 expression plasmids, and the cell lysates were immunoprecipitated with MR14-E5, anti-His, or anti-ITGA2 antibodies. The immunoprecipitates were analyzed by Western blotting with anti-ITGA2 or anti-His antibodies. **g** Overexpression and immunoprecipitation of integrin α3 (ITGA3) with ER7-A7, ET7-A8, anti-His, or anti-ITGA3 antibodies. HEK293T cells were transfected with pcDNA3.1+ or pCMV-FLAG-ITGA3 expression plasmids, and the cell lysates were immunoprecipitated with ER7-A7, ER7-A8, anti-FLAG, or anti-ITGA3 antibodies. The immunoprecipitates were analyzed by Western blotting with anti-ITGA3 or anti-FLAG antibodies. **h** Overexpression and immunoprecipitation of integrin αV (ITGAV) with MR1-B1, anti-FLAG, or anti-ITGAV antibodies. HEK293T cells were transfected with pcDNA3.1+ or pCMV-FLAG-ITGAV expression plasmids, and the cell lysates were immunoprecipitated with MR1-B1, anti-FLAG, or anti-ITGAV antibodies. The immunoprecipitates were analyzed by Western blotting with anti-ITGAV or anti-FLAG antibodies. Preclearing was done with protein G agarose beads and used as a negative control

To identify the target antigens recognized by selected MAbs, biotin-labeled A549 cell lysates were immunoprecipitated with the selected MAbs, and the immunoprecipitates were analyzed by streptavidin-HRP in Western blot analysis. Seven MAbs were able to immunoprecipitate surface molecules with various molecular weights (Fig. 2a–e). Mass spectrometry revealed that MR14-E5 and MR1-B1 recognized integrin α2 (ITGA2) and integrin αV (ITGAV), respectively, while both ER7-A7 and ER7-A8 recognized integrin α3 (ITGA3) (Additional file 1: Figure S5, S6, S7). To demonstrate that MR14-E5 recognizes integrin α2, the His-tagged ITGA2 expression plasmid was transfected into HEK293T cells, and the cell lysates were subjected to immunoprecipitation with MR14-E5, polyclonal anti-integrin α2 or anti-His antibodies, and the immunoprecipitates were detected with anti-integrin α2 or anti-His antibodies. All immunoprecipitates were detected with anti-integrin α2 or anti-His antibodies (Fig. 2f), indicating that the MR14-E5 antigen is integrin α2 indeed. To demonstrate that both ER7-A7 and ER7-A8 recognize integrin α3, the FLAG-tagged ITGA3 expression plasmid was transfected into HEK293T cells, and the cell lysates were subjected to immunoprecipitation with ER7-A7, ER7-A8, polyclonal anti-integrin α3, and anti-FLAG antibodies, and immunoprecipitates were detected with anti-integrin α3 or anti-FLAG antibodies. All immunoprecipitates were detected with anti-integrin α3 or anti-FLAG antibodies (Fig. 2g), indicating that both ER7-A7 and ER7-A8 antigens are integrin α3 indeed. To demonstrate that MR1-B1 recognizes integrin αV, the FLAG-tagged ITGAV expression plasmid was transfected into HEK293T cells, and the cell lysates were subjected to immunoprecipitation with MR1-B1, polyclonal anti-integrin αV, and anti-FLAG antibodies, and the immunoprecipitates were detected with anti-integrin αV or anti-FLAG antibodies. All immunoprecipitates were detected with anti-integrin αV or anti-FLAG antibodies (Fig. 2h), indicating that the MR1-B1 antigen is integrin αV indeed. Shown in Fig. 1, the results also suggest that integrin α3 recognized with ER7-A7 and ER7-A8 and integrin αV recognized with MR1-B1 are upregulated during osteogenic differentiation of hMSCs, while integrin α2 recognized with MR14-E4 is downregulated during osteogenic differentiation of hMSCs.

### Integrin α2 is decreased during osteogenic differentiation of hMSCs while integrin α3 and αV are increased

**Fig. 3:**
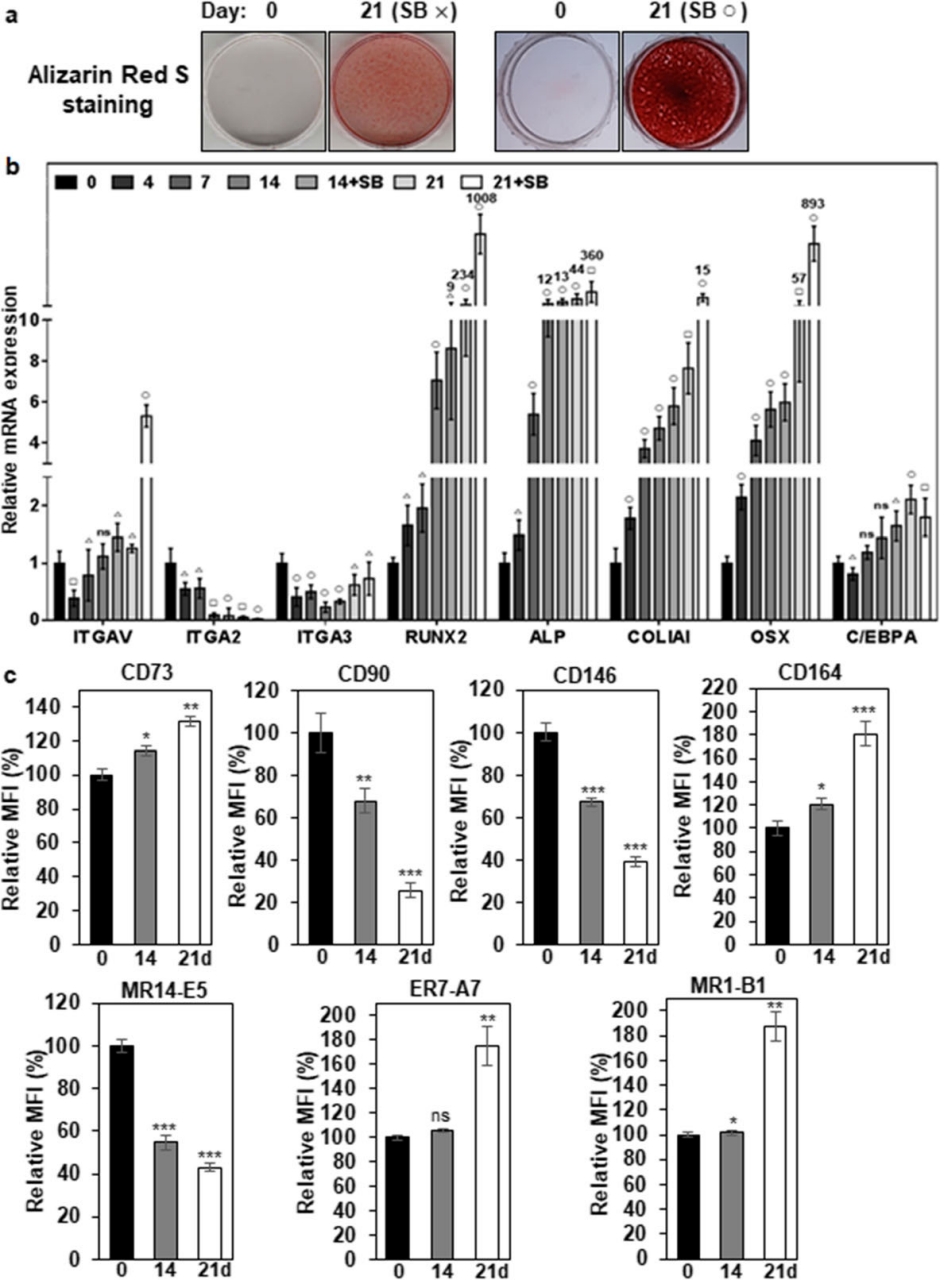
Expression changes of integrin αV, α2, α3, and osteogenic markers during differentiation of hMSCs. **a** Alizarin Red S staining of OBs in differentiated hMSCs. hMSCs were incubated for 21 days with ODM (SB×). To inhibit TGF-β1 signaling that originated from the culture medium, SB431542 was also included in ODM (SB○) after 7 days of osteogenic differentiation. Calcium deposition was visualized as red color after the cells were stained with Alizarin Red S. **b** Expression changes of integrins and osteogenic markers during osteogenic differentiation of hMSCs. Integrins (MR14-E5, ER7-A7, MR1-B1), osteogenic markers (Runx2, ALP, COL1A1, OSX), and adipogenic marker (C/EBPA) were analyzed in differentiated hMSCs by quantitative PCR. Values are depicted as a mean fold change in gene expression (2^−ΔΔCT^) of differentiated hMSCs at the indicated days compared to hMSCs at day 0. △, *p* < 0.05; □, *p* < 0.01; ○, *p* < 0.005; ns, not significant. **c** Expression changes of integrins and hMSC/OB surface markers during osteogenic differentiation of hMSCs. hMSCs were incubated for 21 days with ODM, and SB431542 was added to ODM after 7 days of osteogenic differentiation. Integrins (α2, α3, αV) and hMSC/OB surface markers (CD73, CD90, CD146, and CD164) were analyzed in differentiated hMSCs by flow cytometry. Values are depicted as a relative mean fluorescence intensity (MFI) of differentiated hMSCs at the indicated days compared to hMSCs cultured in normal medium. **, *p* < 0.01; ***, *p* < 0.005; ns, not significant

Next, we examined expression pattern of integrin α2, α3, and αV during differentiation of hMSCs. hMSCs were cultured for 21 days under conditions that promote osteogenic differentiation. SB431542, a specific inhibitor of TGF-β receptor kinase, was added to ODM after 7 days of osteogenic differentiation because it enhances osteogenic differentiation of some somatic stem cells [40, 41]. Although osteogenic differentiation of hMSCs was observed in the absence of SB431542, osteogenic differentiation of hMSCs was obvious in the presence of SB-431542 at 21 days of osteogenic differentiation by increased calcium deposition (Fig. 3a) and increased expression of Runx2 (1008-fold), alkaline phosphatase (ALP, 360-fold), collagen type I (COL1A1, 15-fold), and osterix (OCX, 893-fold) (Fig. 3b). However, the adipogenic marker C/EBPA was slightly increased (1.8-fold) at 21 days of osteogenic differentiation (Fig. 3b). The obvious increase of the early OB markers (Runx2, ALP, OSX) suggests that the differentiated hMSCs are mainly at the stage of early OBs [42]. Under the circumstances, the expression level of integrin α2 mRNA (ITGA2) was gradually decreased to 4.6-fold during osteogenic differentiation of hMSCs (Fig. 3b). The expression level of integrin α3 mRNA (ITGA3) was initially decreased until 14 days of osteogenic differentiation of hMSCs, but it was slightly upregulated up to approximately 0.7-fold at 21 days of osteogenic differentiation (Fig. 3b). Interestingly, the expression level of integrin αV mRNA (ITGAV) was slightly increased up to approximately 1.5-fold at 14 days of osteogenic differentiation of hMSCs and then increased up to approximately 5.3-fold at 21 days of osteogenic differentiation in the presence of SB431542 (Fig. 3b). The results suggest that the increased expression of integrin αV is needed for the maturation of OBs.

Next, we examined the cell surface expression of integrin α2, α3 and αV by flow cytometry during osteogenic differentiation of hMSCs. CD73, CD90, CD146, and CD164 expression were also examined because they are also known as hMSC and/or osteoprogenitor markers [37, 43–46]. CD90 and CD146 were drastically downregulated during osteogenic differentiation of hMSCs (Fig. 3c and Additional file 1: Figure S8). Interestingly, CD73 and CD164 were upregulated during osteogenic differentiation of hMSCs (Fig. 3c and Additional file 1: Figure S8). Under the circumstances, the cell surface expression of integrin α2 (recognized by MR14-E5) was drastically downregulated during osteogenic differentiation of hMSCs (Fig. 3c and Additional file 1: Figure S8). However, the cell surface expression of integrin α3 (recognized by ER7-A7) was slightly (106%) upregulated at 14 days of osteogenic differentiation and then further upregulated up to approximately 176% at 21 days of osteogenic differentiation (Fig. 3c and Additional file 1: Figure S8). The cell surface expression of integrin αV (recognized by MR1-B1) was also upregulated up to approximately 187% at 21 days of osteogenic differentiation (Fig. 3c and Additional file 1: Figure S8), which is also consistent with increased ITGAV mRNA expression (Fig. 3b). However, integrins, including integrin α3 and αV, were drastically downregulated during adipogenic differentiation of hMSCs like the surface markers of hMSC/osteoprogenitors (Additional file 1 : Figure S9), suggesting that increased expression of integrin α3 and αV is an osteogenic-specific property of hMSCs. To further investigate whether increased expression of α3 and αV is due to the effects of SB431542 on osteogenic differentiation of hMSCs, hMSCs were also analyzed in the absence and presence of SB431542 during osteogenic differentiation of hMSCs. Osteogenic differentiation of hMSCs was observed even without SB-431542 at 21 days of osteogenic differentiation by increased calcium deposition (Additional file 1: Figure S10a). Under the same circumstances, the surface expression of hMSC/OB markers did not show a significant difference between the presence and absence of SB431542 (Additional file 1: Figure S10b). The surface expression of integrin α3 and αV also showed similar expression profiles between the presence and absence of SB431542 (Additional file 1: Figure S10b). The results suggest that increased expression of integrin α3 and αV is the outcomes of osteogenic differentiation of hMSCs rather than the specific effects of SB431542 treatment.

### Integrin α3 and αV are upregulated in hPDLCs and hDPCs upon osteogenic differentiation

**Fig. 4:**
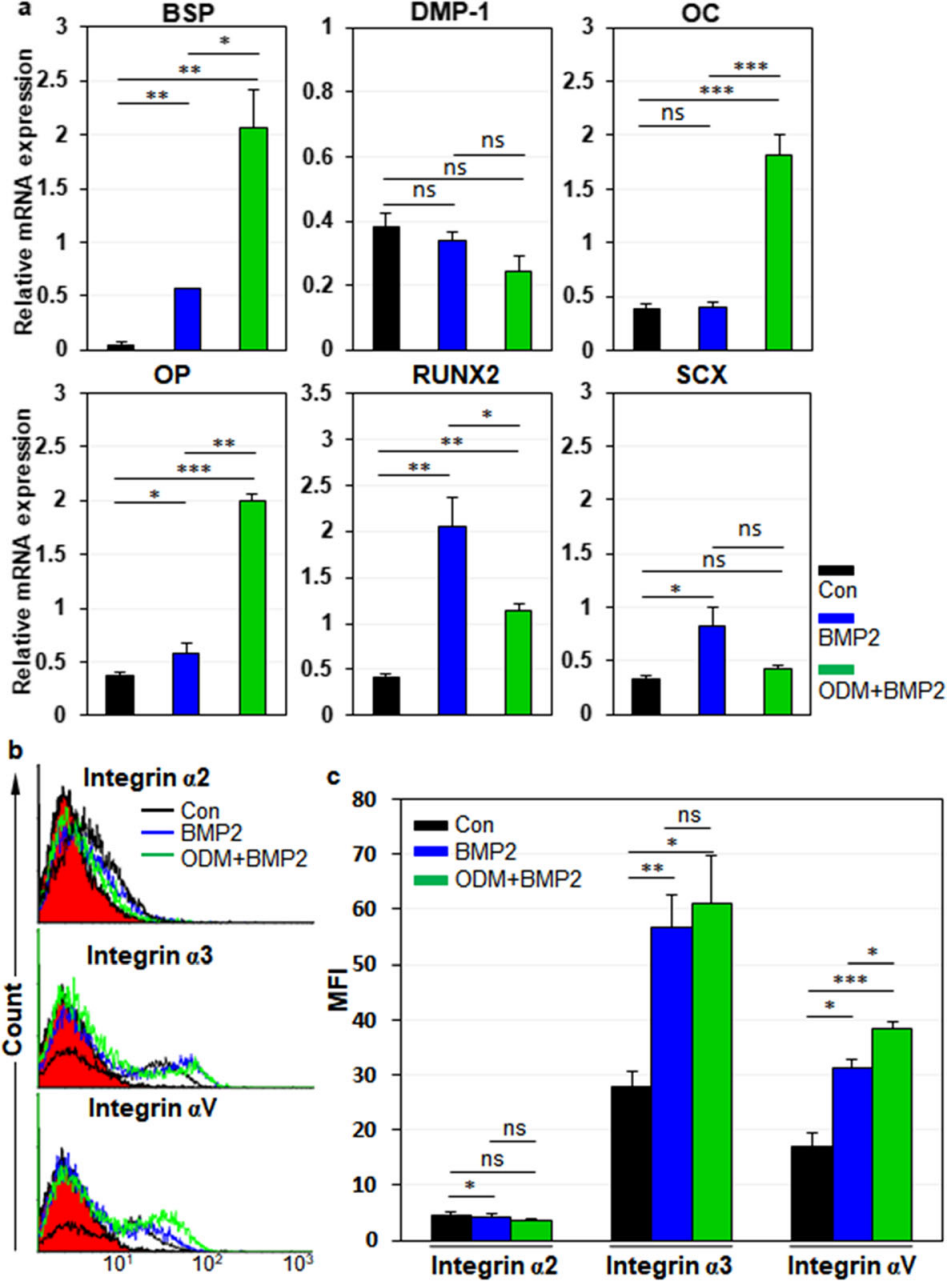
Expression changes of integrin αV, α2, and α3 during osteogenic differentiation of hPDLCs. hPDLCs were incubated for 7 days with BMP-2, followed by treatment with ODM for additional 7 days for mineralization. **a** Osteogenic differentiation of hPDLCs. Osteogenic and odontogenic markers (BSP, DMP-1, OC, OP, Runx2) and ligamentogenic marker (SCX) were analyzed by quantitative PCR. **b**, **c** Expression changes of integrins during osteogenic differentiation of hPDLCs. Cell surface expression of integrins in osteogenic hPDLCs was analyzed by flow cytometry. Values are depicted as MFIs at the indicated culture conditions. Black, incubation in media without BMP-2 treatment; blue, incubation in media with BMP-2 treatment; green, incubation in ODMs with BMP-2. *, *p* < 0.05; **, *p* < 0.01; ***, *p* < 0.005; ns, not significant

**Fig. 5:**
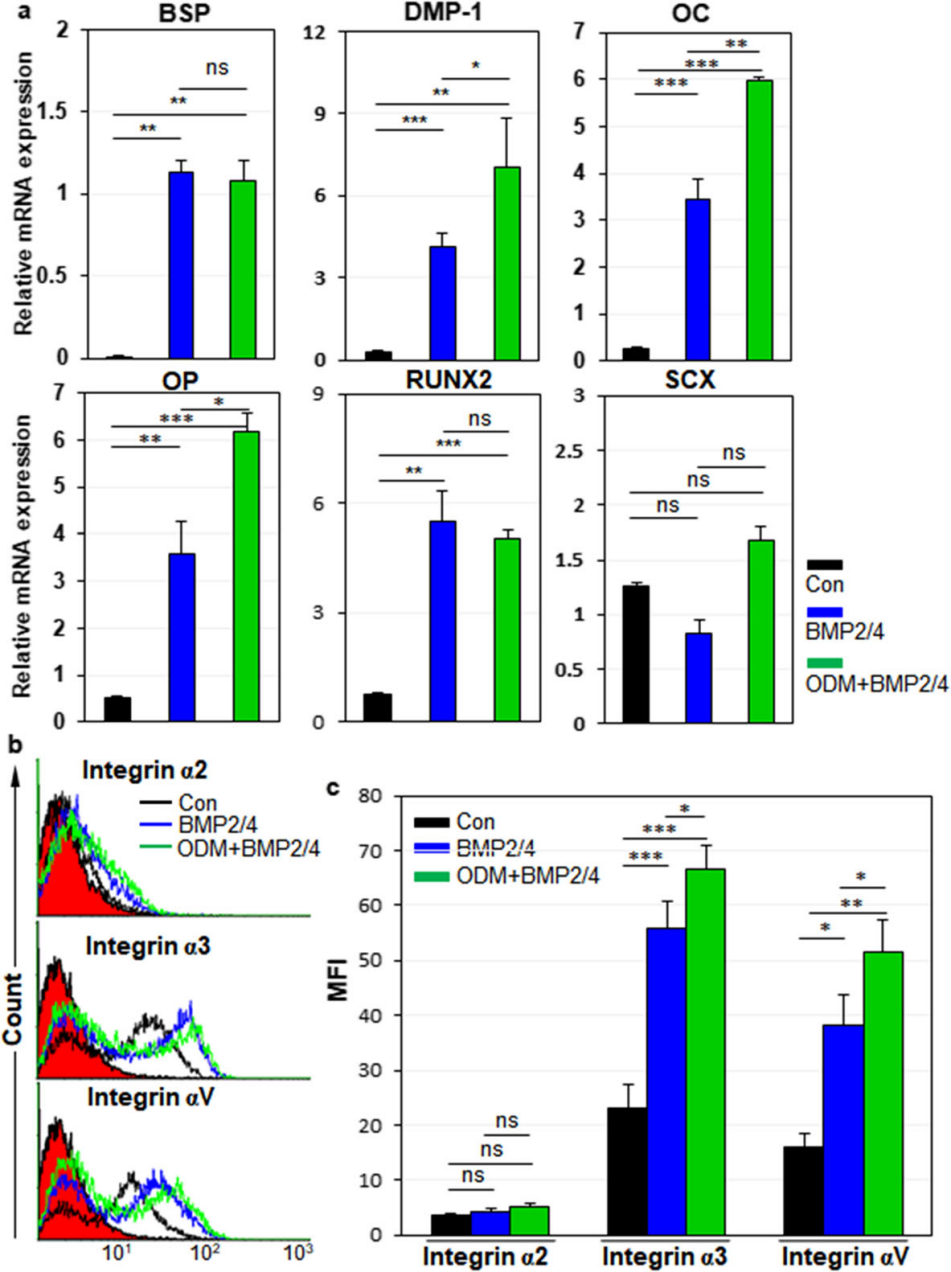
Expression changes of integrin αV, α2, and α3 during osteogenic differentiation of hDPCs. hDPCs were incubated for 7 days with BMP-2/4, followed by treatment with ODM for additional 7 days for mineralization. **a** Osteogenic differentiation of hDPCs. Osteogenic and odontogenic markers (BSP, DMP-1, OC, OP, Runx2) and ligamentogenic marker (SCX) were analyzed by quantitative PCR. **b**, **c** Expression changes of integrins during osteogenic differentiation of hDPCs. Cell surface expression of integrins in the osteogenic hDPCs was analyzed by flow cytometry. Values are depicted as MFIs at the indicated culture conditions. Black, incubation in media without BMP-2/4 treatment; blue, incubation in media with BMP-2/4 treatment; green, incubation in ODMs with BMP-2/4. *, *p* < 0.05; **, *p* < 0.01; ***, *p* < 0.005; ns, not significant

Next, we examined the expression dynamics of integrin α2, α3, and αV in hPDLCs during osteogenic differentiation of hPDLCs. For osteoblastic differentiation of hPDLCs, cells were treated with BMP-2 or ODM-plus BMP-2 as described previously [30]. Relative mRNA expression of bone sialoprotein (BSP), osteocalcin (OC), osteopontin (OP), and Runx2 were increased in BMP-2 and ODM-plus BMP-2 treatment, although relative mRNA expression of ligamentogenic marker scleraxis (SCX) was not altered in ODM-plus BMP-2 treatment (Fig. 4a). Under the circumstances, integrin α2 expression was slightly downregulated in BMP-2 treatment but not altered in ODM-plus BMP-2 treatment (Fig. 4b, c). However, integrin α3 and αV expression were drastically upregulated in BMP-2 treatment and further increased in ODM-plus BMP-2 treatment (Fig. 4b, c). The same experiments were also done with hDPCs. For osteoblastic and odontoblastic differentiation of hDPCs, cells were treated with BMP-2/4 or ODM-plus BMP-2/4 as described previously [31]. Relative mRNA expression of BSP, dentin matrix protein-1 (DMP-1), OC, OP, and Runx2 were increased in BMP-2/4 treatment (Fig. 5a). Relative mRNA expression of DMP-1, OC, and OP were further increased in ODM-plus BMP-2/4 treatment, while relative mRNA expression of BSP and Runx2 were just maintained in ODM-plus BMP-2/4 treatment (Fig. 5a). SCX was not altered in both culture conditions (Fig. 5a). Under the circumstances, Integrin α2 expression was not altered in both BMP2/4 and ODM-plus BMP-2/4 treatment (Fig. 5b, c). Again, integrin α3 and αV expression were drastically upregulated in BMP2/4 treatment and further increased in ODM-plus BMP-2/4 treatment (Fig. 5b, c). Taken together, the results suggest that the cell surface expression of integrin α3 and αV is upregulated in hPDLCs and hDPCs upon osteogenic differentiation of them. The results also suggest that integrin α3 and αV expression is commonly upregulated in hMSCs, hPDLCs, and hDPCs upon osteogenic differentiation of them.

### Integrin αV-high hMSCs have a greater osteogenic potential than integrin αV-low hMSCs

**Fig. 6:**
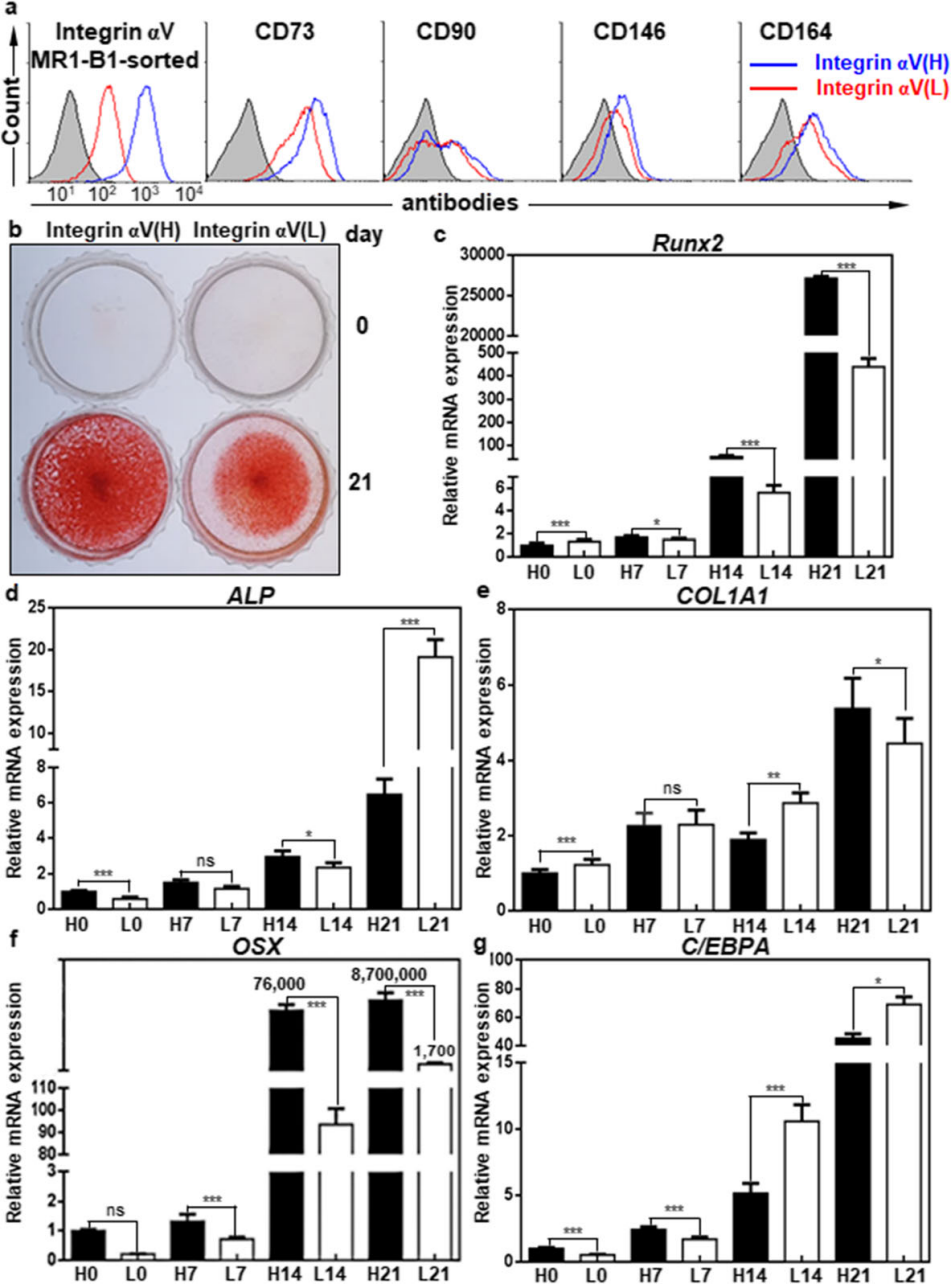
Integrin αV-high hMSCs have a greater osteogenic potential than integrin αV-low hMSCs. **a** Flow cytometric analysis of hMSCs with MR1-B1, anti-CD73, anti-CD90, anti-CD146, and anti-CD164 antibodies in integrin αV-high and αV-low hMSCs after MR1-B1-based magnetic cell sorting. **b** Alizarin Red S staining of OBs in integrin αV-high and αV-low hMSCs after being cultured in ODM for 21 days. To inhibit TGF-β1 signaling that originated from the culture medium, SB431542 was added to ODM after 7 days of osteogenic differentiation. Calcium deposition was visualized as red color after the cells were stained with Alizarin Red S. **c–g** Expression changes of osteogenic and adipogenic markers during osteogenic differentiation of hMSCs. Osteogenic markers (Runx2, ALP, COL1A1, OSX) and adipogenic marker (C/EBPA) were analyzed at 0, 7, 14, and 21 days of osteogenic differentiation in integrin αV-high (H0, H7, H14, H21) and αV-low (L0, L7, L14, L21) hMSCs by quantitative PCR. Values are depicted as a mean fold change in gene expression (2^−ΔΔCT^) of differentiated hMSCs at the indicated days compared to integrin αV-high hMSCs at day 0 (H0). *, *p* < 0.05; **, *p* < 0.01; ***, *p* < 0.005; ns, not significant

Next, we examined the osteogenic potential of integrin αV-high and -low hMSCs after MR1-B1-based magnetic cell sorting. Both integrin αV-high and αV-low hMSCs were cultured in ODM for 21 days. As shown in Fig. 3, SB431542 was added to ODM after 7 days of osteogenic differentiation. All of the OB surface markers (CD73, CD90, CD146, and CD164) were slightly upregulated in integrin αV- high hMSCs as compared to those of integrin αV-low hMSCs (Fig. 6a). ARS staining revealed that integrin αV-high hMSCs had a greater osteogenic potential than integrin αV-low hMSCs (Fig. 6b). The expression levels of Runx2 and OSX were drastically upregulated to approximately 2.7 × 10^4^- and 8.7 × 10^6^-fold, respectively, during osteogenic differentiation, and they were approximately 62-fold and 5023-fold higher, respectively, in integrin αV-high hMSCs than in integrin αV-low hMSCs at 21 days of osteogenic differentiation (Fig. 6c, f). The expression level of COL1A1, a late OB marker, was gradually upregulated and was also higher at 21 days of osteogenic differentiation in integrin αV-high hMSCs than in integrin αV-low hMSCs, although the expression level of ALP was downregulated in integrin αV-high hMSCs than in integrin αV-low hMSCs (Fig. 6d, e). The expression level of C/EBPA, an adipogenesis marker, was also gradually upregulated but significantly lower at 21 days of osteogenic differentiation in integrin αV-high hMSCs than in integrin αV-low hMSCs (Fig. 6d, g). Thus, the expression levels of key osteogenic transcription factors (Runx2, OSX) and COL1A1 were upregulated in integrin αV-high hMSCs whereas the expression level of adipogenic transcription factor C/EBPA was downregulated in integrin αV-high hMSCs upon osteogenic differentiation of hMSCs. The results suggest that integrin αV-high hMSCs have a greater osteogenic potential than integrin αV-low hMSCs.

### Integrin αV induction is a good indicator of OB differentiation

**Fig. 7:**
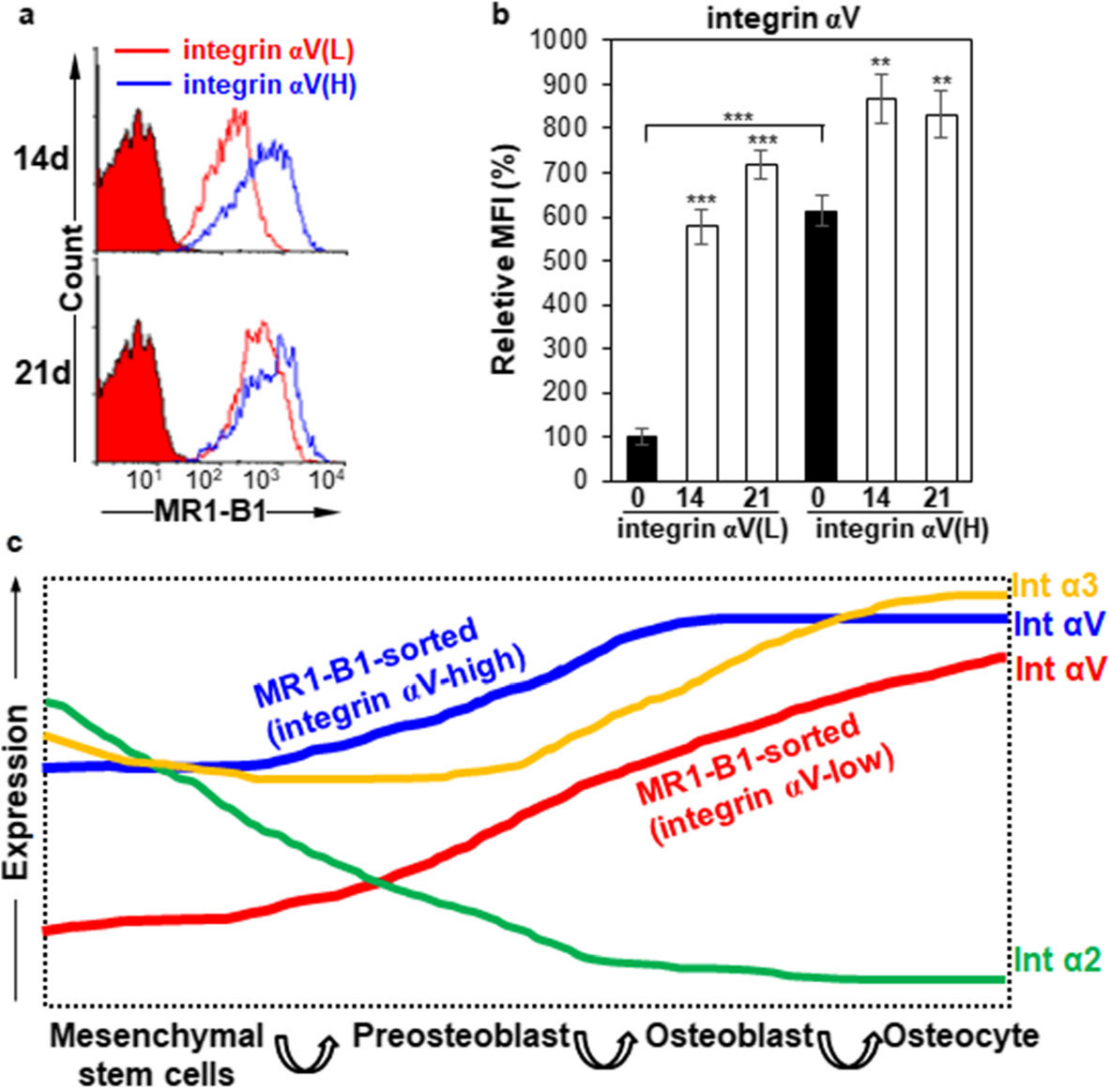
Expression changes of integrin αV during osteogenic differentiation of hMSCs after cell sorting. **a** Flow cytometric analysis of integrin αV-high and αV-low hMSCs after MR1-B1-based magnetic cell sorting. hMSCs were cultured in ODM for 21 days after MR1-B1-based magnetic cell sorting. To inhibit TGF-β1 signaling that originated from the culture medium, SB431542 was added to ODM after 7 days of osteogenic differentiation. The cells were detached and analyzed at days 14 and 21 by flow cytometry. **b** Statistic analysis of **a**. Values are depicted as relative MFIs of sorted hMSCs at the indicated days compared to sorted hMSCs cultured in normal medium. **, *p* < 0.01; ***, *p* < 0.005. **c** Proposed model of expression dynamics of integrin α2, α3, and αV during osteogenic differentiation of hMSCs. Upon osteogenic differentiation of hMSCs, integrin α2 expression is rapidly downregulated and integrin α3 and αV expression is gradually upregulated upon osteogenic differentiation of hMSCs. Integrin αV expression is more dramatically induced even in integrin αV-low hMSCs, suggesting that integrin αV induction is needed for OB differentiation of hMSCs

Interestingly, all the osteogenic markers were also gradually upregulated even in integrin αV-low hMSCs during osteogenic differentiation, and ALP was even threefold higher at 21 days of osteogenic differentiation in integrin αV-low hMSCs than in integrin αV-high hMSCs (Fig. 6c–g). Therefore, we compared the expression changes of integrin αV between integrin αV-high and αV-low hMSCs by flow cytometry. Integrin αV expression was drastically recovered (approximately sevenfold increase) at 21 days of osteogenic differentiation in integrin αV-low hMSCs (Fig. 7a, b). As shown in Fig. 6, the osteogenic early and late markers Runx2, ALP, OSX, and COL1A1 were also highly increased at 21 days of osteogenic differentiation in integrin αV-low hMSCs, as compared to day 0, suggesting that the increased integrin αV expression is closely associated with osteogenic induction. Consistent with this notion, a substantial amount of ARS staining was observed in integrin αV-low hMSCs at 21 days of osteogenic differentiation (Fig. 6b). Taken together, the results suggest that integrin αV induction is a good indicator of the early and late stages of OB differentiation even in integrin αV-low hMSCs.

## Discussion

Osteogenesis is driven by a sequential biological processes initiated by the recruitment of MSCs to bone formation sites, subsequent proliferation, lineage commitment into pre-osteoblast and OB, expression of lineage-specific markers, extracellular matrix secretion, and mineralization [47]. There are many progressive changes in osteogenic lineage cells during osteogenic differentiation of MSCs but there are no definitive surface markers for OBs except for alkaline phosphatase [42]. We postulated that some of the cell surface molecules on TGF-β1-treated cancer cells closely resemble the surface molecules on OBs because TGF-β1 treatment enriches the population of osteogenic progenitors and activates the early differentiation of OBs by inhibiting late differentiation of OBs [9, 10]. To identify novel cell surface molecules on EMT-phenotypic cancer cells, in the beginning, we generated a panel of MAbs specific to the cell surface-expressed epitopes on TGF-β1-treated cancer cells by immunizing TGF-β1-treated A549 cells in mice. Compared with conventional peptide-based approaches, establishing MAbs against the surface molecules on intact cells expands the repertoire of MAbs against the conformation and post-translational modification of these surface molecules [48]. Therefore, we postulated that the MAbs would be useful tools to find novel surface markers or epitopes on TGF-β1-regulated OB cells. Nine MAbs were selected from the generated MAbs because they bound to hMSCs and hFOBs, and the cell surface expression of cognate antigens showed up- or downregulation during osteogenic differentiation of hMSCs (Fig. 1, Table 1). The cell surface expression of cognate antigens was also upregulated or downregulated in Runx2 knockdown osteosarcoma cells (Fig. 1, Table 1). Therefore, we thought that the MAbs were responsible for physiological recognition of cognate antigens during osteogenic differentiation because they were generated by immunizing intact cells. Immunoprecipitation followed by LC-MS/MS identified the antigens of 4 MAbs (MR14-E5, ER7-A7, ER7-A8, and MR1-B1) as integrin α2, α3, α3, and αV, respectively. By using MR1-B1, we found that integrin αV-high hMSCs have a greater osteogenic potential than integrin αV-low hMSCs. The results also suggest that integrin αV induction is a good indicator of the early and late stages of OB differentiation because the cell surface expression of integrin αV was induced from integrin αV-low hMSCs upon osteogenic differentiation of hMSCs. The increased expression of integrin αV was also obvious in hPDLCs and hDPCs during osteogenic differentiation of them. These findings suggest that integrin αV recognized by MR1-B1 can serve as a definitive cell surface marker of OB differentiation. A proposed model for expression dynamics of integrin α2, α3, and αV upon osteogenic differentiation of hMSCs is presented (Fig. 7c).

To accomplish bone formation and regeneration, it is important to investigate cell-matrix interaction mediated by various integrins of hMSC-derived OBs. Integrins are cell surface receptors which can bind extracellular matrix (ECM) components, membrane-bound cell surface molecules, or soluble extracellular ligands for cell migration, differentiation, survival, and proliferation [49]. The present study showed that cell surface expression of integrin α2, α3, and αV was observed in undifferentiated hMSCs, consistent with previous studies [49] (Additional file 1: Figure S3). The cell surface expression of integrin α2 was drastically downregulated after 14 days of osteogenic differentiation of hMSCs (Fig. 3c). The cell surface expression of integrin α2 was not observed in hPDLCs and hDPCs even in ODM (Figs. 4, 5). Furthermore, the drastic downregulation of integrin α2 was also observed during adipogenic differentiation of hMSCs (Additional file 1: Figure S9), suggesting that integrin α2 is a cell surface marker for undifferentiated hMSCs. Consistent with this notion, integrin α2 is critical for the survival of hMSCs via collagen I binding [50, 51]. The cell surface expression of integrin α3β1 was not observed in bone marrow-derived hMSCs [52], but integrin α3 recognized with ER7-A7 and ER7-A8 was readily detected in bone marrow-derived hMSCs (Fig. 1 and Additional file 1: Figure S3). The cell surface expression of integrin α3 was downregulated during adipogenic differentiation of hMSCs (Additional file 1: Figure S9), but it was obviously upregulated during osteogenic differentiation of hMSCs (Fig. 3), suggesting that increased expression of integrin α3 is needed for the later stage of osteogenic differentiation. Interestingly, the cell surface expression of integrin α3 was drastically induced in hPDLCs and hDPCs during osteogenic differentiation of them (Figs. 4, 5), suggesting that integrin α3 expression is needed for osteogenic differentiation of hPDLCs and hDPCs as well. Consistent with the results, integrin α3 is expressed by OBs actively synthesizing bone and some of the OB lining cells [53].

The cell surface expression of integrin αV was further increased in bone marrow-derived hMSCs during osteogenic differentiation of bone marrow-derived hMSCs (Figs. 3, 7). The same results were also observed in hPDLCs and hDPCs, suggesting that integrin αV is generally increased during osteogenic differentiation of hMSCs, hPDLCs, and hDPCs. This observation drew our interest because little is known about the role of integrin αV in hMSC commitment to OB lineage. When integrin αV-low hMSCs were subjected to osteogenic differentiation after MR1-B1-based magnetic cell sorting, increased expression of integrin αV was obvious after 14 days of osteogenic differentiation of hMSCs (Fig. 7). It seems that integrin αV induction is needed for the hMSC commitment into OB lineage. Integrin αV expression drives the proliferation and survival of human adipose-derived stem cells (hASCs) through the suppression of p21 and induction of survivin and TAZ [54]. Therefore, it is possible to speculate that integrin αV may drive the survival, proliferation, and differentiation of OBs during osteogenic differentiation of hMSCs. This phenomenon was not observed during adipogenic differentiation of hMSCs (Additional file 1: Figure S9). It may be that this observation is due to differences between hMSCs and hASCs, which remain elusive.

Increased surface expression of integrin αV indicated the hMSC commitment into OB lineage (Figs. 3, 4, 5, 6, 7). The results may indicate that the expression and secretion of ECM, especially integrin αV ligands, such as fibronectin, vitronectin, and osteopontin, may be important for the survival, proliferation, and differentiation of OB during osteogenic differentiation. A previous study showed that the interaction between integrin αVβ1 and connective tissue growth factor (CTGF/CCN2) stimulates OB growth and differentiation via integrin-mediated activation of ERK signaling and an increase in Runx2 binding to the osteocalcin promoter [55]. Integrin αVβ3 is also implicated in osteogenesis of bone marrow-derived hMSCs cultured on fibronectin coated stiff substrates [56]. By using a αVβ3 blocking antibody, another study also confirmed that integrin αVβ3 is responsible for the OB commitment from dental bud stem cells through its interaction with vitronectin and osteopontin [57]. Thus, the present studies including the previous studies indicate that increased expression of integrin αV is needed for the commitment of hMSCs to OB differentiation and maturation.

## Conclusion

In this study, we generated 4 MAbs specific to integrin α2, α3, or αV on bone marrow-derived hMSCs. By using the MAbs, we found that the cell surface expression of integrins fluctuated during osteogenic differentiation of hMSCs depending on the integrin type. Integrin α2 recognized by MR14-E5 was downregulated on hMSCs during osteogenic differentiation, while integrin α3 and αV, recognized by ER7-A7 and MR1-B1, respectively, were upregulated on hMSCs in accordance with upregulation of osteogenic markers. Subsequent studies showed that integrin α3 and αV are also upregulated in hPDLCs and hDPCs during osteogenic differentiation, suggesting that integrin α3 and αV induction is a good indicator of OB differentiation. Cell sorting revealed that key osteogenic induction factors (Runx2, OSX, and COL1A1) and surface markers (CD73, CD90, CD146, and CD164) are higher in integrin αV-high hMSCs than in integrin αV-low hMSCs upon osteogenic differentiation of hMSCs, suggesting that integrin αV-high hMSCs have a greater osteogenic potential than integrin αV-low hMSCs. Cell sorting further revealed that integrin αV expression is more dramatically induced in integrin αV-low hMSCs during osteogenic differentiation of hMSCs. The results suggest that integrin αV induction is a good indicator of the early and late stages of OB differentiation.

## Supplementary information

**Additional file 1: Table S1.** Primer sequences used for quantitative real-time PCR. **Table S2.** Hybridoma clones specific to EMT-phenotypic A549 cells. **Table S3.** Expression profiles of MAb antigens on mesenchymal lineage cells with osteogenic potential. **Figure S1.** TGF-β1-treated A549 cells exhibit EMT phenotype. (a) Morphology of A549 and TGF-β1-treated A549 cells. A549 cells were treated with 5 ng/ml TGF-β1 for 4 days. The scale bar is 200 μm. (b) Expression of EMT markers in TGF-β1-treated A549 cells. TGF-β1-treated A549 cells were analyzed by Western blotting with the indicated antibodies. (c) Cell surface expression of EMT markers on TGF-β1-treated A549 cells. TGF-β1-treated A549 cells were analyzed by flow cytometry with the indicated antibodies. Red-filled histograms represent the isotype controls. **Figure S2.** Screening of hybridoma clones specific to EMT-phenotypic A549 cells. Expression of MAb antigens was analyzed in A549 and TGF-β1-treated A549 cells by flow cytometry with the indicated MAbs. The expression of E-cadherin (E-cad) and N-cadherin (N-cad) was used as controls. Red-filled histograms represent the isotype controls. **Figure S3.** Cell surface expression of target antigens of selected MAbs in mesenchymal stem and progenitor cells with osteogenic potential. Cell surface expression of target antigens of selected MAbs was examined in two human osteogenic progenitor cells (hMSC and hFOB) and two human osteoblastic cancer cell lines (U2OS and SAOS-2) by flow cytometry with the indicated MAbs. Red-filled histograms represent the isotype controls. **Figure S4.** Alizarin Red S staining assay and knockdown efficiency of Runx2 in U2OS cells. (a) Alizarin Red S staining of hMSCs stimulated with ODM. hMSCs were incubated for 12 days with ODM, and calcium deposition and bone nodule were visualized as red color after the cells were stained with Alizarin Red S. The scale bar is 200 μm. (b) Knockdown efficiency of Runx2 in U2OS cells. After transfection of control siRNA or Runx2 siRNA, the expression of Runx2 gene was evaluated by RT-PCR (left panels) and by Western blotting (right panels). GAPDH or β-actin was used as a loading control. **Figure S5.** Mass spectrometric identification of MR14-E5 antigen after immunoprecipitation with ME14-E5. The approximately 150-kDa band from A549 cell lysates was treated with trypsin, and the resulting peptides were analyzed by mass spectrometry. Ten tryptic peptides (underlined) originating from the 150-kDa protein matched the integrin α2 precursor. **Figure S6.** Mass spectrometric identification of ER7-A7 and ER7-A8 antigen after immunoprecipitation with ER7-A8. The approximately 130-kDa band from A549 cell lysates was treated with trypsin, and the resulting peptides were analyzed by mass spectrometry. Five tryptic peptides (underlined) originating from the 130-kDa protein matched the integrin α3 preproprotein. **Figure S7.** Mass spectrometric identification of MR1-B1 antigen after immunoprecipitation with MR1-B1. The approximately 130-kDa band from A549 cell lysates was treated with trypsin, and the resulting peptides were analyzed by mass spectrometry. Five tryptic peptides (underlined) originating from the 130-kDa protein matched the integrin αV isoform 1 preproprotein. **Figure S8.** Expression changes of integrins and hMSC/OB surface markers upon osteogenic differentiation of hMSCs. hMSCs were incubated for 14, 21 days with ODM, and SB431542 was added to ODM after 7 days of the osteogenic differentiation. Integrins (α2, α3, αV), hMSC/OB surface markers (CD73, CD90, CD146 and CD164) were analyzed in undifferentiated (normal growth medium) and differentiated hMSCs (ODM) by flow cytometry. Red-filled histograms represent isotype controls. **Figure S9.** Expression changes of integrin αV, α2, α3 and osteogenic markers during adipogenic differentiation of hMSCs. (a) Oil Red O staining of adipocytes in differentiated hMSCs. hMSCs were incubated for 21 days with ADM. Lipid content was visualized as red color after the cells were stained with Oil Red O. (b) Expression changes of integrins and hMSC/OB surface markers during adipogenic differentiation of hMSCs. Integrins (MR14-E5, ER7-A7, MR1-B1) and hMSC/OB surface markers (CD73, CD90, CD146 and CD164) were analyzed in differentiated hMSCs by flow cytometry. Values are depicted as a relative MFI of differentiated hMSCs at the indicated days compared to hMSCs at day 0. **, *p* < 0.01; ***, *p* < 0.005. **Figure S10.** Expression changes of integrins and hMSC/OB surface markers upon osteogenic differentiation of hMSCs in the absence and presence of SB431542. (a) Alizarin Red S staining of hMSCs stimulated with ODM. hMSCs were incubated for 21 days with ODM in the absence and presence of SB431542. SB431542 was included in ODM after 14 days of osteogenic differentiation and calcium deposition and bone nodule were visualized as red color after the cells were stained with Alizarin Red S. (b) Flow cytometric analysis of hMSCs cultured in ODM in the absence and presence of SB431542. hMSCs were incubated for 7, 14, 21 days with ODM, and SB431542 was added to ODM after 14 days of osteogenic differentiation. Integrins (α2, α3, αV), hMSC/OB surface markers (CD73, CD90, CD146 and CD164) were analyzed by flow cytometry. Red-filled histograms represent isotype controls.

## Data Availability

The datasets used and analyzed during the current study are available from the corresponding author on reasonable request.

## Abbreviations

hMSC: Human mesenchymal stem cell
MAb: Monoclonal antibody
OB: Osteoblast
TGF-β1: Transforming growth factor-β1
BMP: Bone morphogenetic protein
ODM: Osteogenic differentiation medium
hPDLCs: Human periodontal ligament cells
hDPCs: Human dental pulp cells
qPCR: Quantitative polymerase chain reaction
ARS: Alizarin Red S
Runx2: Runt-related transcription factor 2
EMT: Epithelial-mesenchymal transition
NSCLC: Non-small cell lung carcinoma cell
bFGF: Basic fibroblast growth factor
FBS: Fetal bovine serum
PBMC: Peripheral blood mononuclear cell
FITC: Fluorescein isothiocyanate
PE: Phycoerythrin
PI: Propidium iodide
IP: Immunoprecipitation
SDS-PAGE: Sodium dodecyl sulfate-polyacrylamide gel electrophoresis
SA-HRP: Streptavidin-horse radish peroxidase
TBST: Tris-buffered saline with Tween-20
ADM: Adipogenic differentiation medium
RT-PCR: Reverse transcription-polymerase chain reaction
ALP: Alkaline phosphatase
COL1A1: Collagen type I
OCX: Osterix
ITGA: Integrin α
BSP: Bone sialoprotein
OC: Osteocalcin
OP: Osteopontin
SCX: Scleraxis
DMP-1: Dentin matrix protein-1
ECM: Extracellular matrix
