# Supplementary material for: Expression dynamics of integrin α2, α3, and αV upon osteogenic differentiation of human mesenchymal stem cells
Source: Stem Cell Res Ther. 2020 Jun 3;11:210. doi: 10.1186/s13287-020-01714-7 (PMC7268774; doi:10.1186/s13287-020-01714-7)
Supplement: Supplementary file 1 — Additional file 1: Table S1. Primer sequences used for quantitative real-time PCR. Table S2. Hybridoma clones specific to EMT-phenotypic A549 cells. Table S3. Expression profiles of MAb antigens on mesenchymal lineage cells with osteogenic potential. Figure S1. TGF-β1-treated A549 cells exhibit EMT phenotype. (a) Morphology of A549 and TGF-β1-treated A549 cells. A549 cells were treated with 5 ng/ml TGF-β1 for 4 days. The scale bar is 200 μm. (b) Expression of EMT markers in TGF-β1-treated A549 cells. TGF-β1-treated A549 cells were analyzed by Western blotting with the indicated antibodies. (c) Cell surface expression of EMT markers on TGF-β1-treated A549 cells. TGF-β1-treated A549 cells were analyzed by flow cytometry with the indicated antibodies. Red-filled histograms represent the isotype controls. Figure S2. Screening of hybridoma clones specific to EMT-phenotypic A549 cells. Expression of MAb antigens was analyzed in A549 and TGF-β1-treated A549 cells by flow cytometry with the indicated MAbs. The expression of E-cadherin (E-cad) and N-cadherin (N-cad) was used as controls. Red-filled histograms represent the isotype controls. Figure S3. Cell surface expression of target antigens of selected MAbs in mesenchymal stem and progenitor cells with osteogenic potential. Cell surface expression of target antigens of selected MAbs was examined in two human osteogenic progenitor cells (hMSC and hFOB) and two human osteoblastic cancer cell lines (U2OS and SAOS-2) by flow cytometry with the indicated MAbs. Red-filled histograms represent the isotype controls. Figure S4. Alizarin Red S staining assay and knockdown efficiency of Runx2 in U2OS cells. (a) Alizarin Red S staining of hMSCs stimulated with ODM. hMSCs were incubated for 12 days with ODM, and calcium deposition and bone nodule were visualized as red color after the cells were stained with Alizarin Red S. The scale bar is 200 μm. (b) Knockdown efficiency of Runx2 in U2OS cells. After transfection of control siRNA or [file 13287_2020_1714_MOESM1_ESM.docx]

**Expression dynamics of integrin α2, α3 and αV upon osteogenic differentiation of human mesenchymal stem cells**

Hyun Min Lee^1^, Se-Ri Seo^1^, Jeeseung Kim^1^, Min Kyu Kim^1^, Hyosun Seo^1^, Kyoung Soo Kim^2^, Young-Joo Jang^3^ and Chun Jeih Ryu^1^

^1^Institute of Anticancer Medicine Development, Department of Integrative Bioscience and Biotechnology, Sejong University, Seoul 05006, Korea. ^2^Department of Clinical Pharmacology and Therapeutics, Kyung Hee University School of Medicine, Seoul 02447, Korea. ^3^Department of Nanobiomedical Science, BK21 PLUS NBM Global Research Center for Regenerative Medicine, College of Dentistry, Dankook University, Cheonan 330-714, Korea

Correspondence to Chun Jeih Ryu, Department of Integrative Bioscience and Biotechnology, Sejong university, 209 Neungdong-ro, Gwangjin-gu, Seoul 05006, Korea, Telephone: 82-2-3408-3718; FAX:82-2-3408-4334; e-mail:[cjryu@sejong.ac.kr](mailto:cjryu@sejong.ac.kr), or to Young-Joo Jang, Department of Nanobiomedical Science, BK21 PLUS NBM Global Research Center for Regenerative Medicine, College of Dentistry, Dankook University, Cheonan 330-714, Korea, Telephone:82-41-550-1936; FAX:82-41-557-9676; email: [yjjang@dankook.ac.kr](mailto:yjjang@dankook.ac.kr).

**Additional file1**

**Table S1. Primer sequences used for quantitative real-time PCR**

| Primer sequences for gene expression analysis in hMSCs and U2OS cells | | |
| --- | --- | --- |
| Gene | Forward primer | Reverse primer |
| ITGA2 | 5’- CCTTGAAGCCTATTCTGAGACTGCC | 5’-AATTCCAGTGTTGTATGCACTTTCCC |
| ITGA3 | 5’-ACTGTGAAGGCACGAGTGTGGAAC | 5’-ATGCTGGTTCGGAGGAATAGGG |
| ITGAV | 5’-GGATTGTTGCTACTGGCTGTTTTGG | 5’-TCCCTTTCTTGTTCTTCTTGAGGTGG |
| RUNX2 | 5’-GACCAGTCTTACCCCTCCTACC | 5’-CTGCCTGGCTCTTCTTACTGAG |
| ALP | 5’-ACTCCCACTTCATCTGGAACC | 5’-CCTGTTCAGCTCGTACTGCAT |
| Col1A1 | 5’-GAGAGGAAGGAAAGCGAGGAG-3’ | 5’-GGGACCAGCAACACCATCT |
| OSX | 5’-GCCAGAAGCTGTGAAACCTC-3’ | 5’-GCTGCAAGCTCTCCATAACC |
| CEBPA | 5’-GGTGGACAAGAACAGCAACG | 5’-CGGTCATTGTCACTGGTCAG |
| GAPDH | 5’ -ACAGCGACACCCACTCCTCC | 5’-GAGGTCCACCACCCTGTTGC |
| Primer sequences for gene expression analysis in hPDLSCs and hDPSCs | | |
| BSP | 5’-ACCGAGCCTATGAAGATGA | 5’-CTTCCTGAGTTGAACTTCGA |
| DMP-1 | 5’-ACTCTCAAGAAGACAGCAA | 5’-GACTCACTCACCACCTCT |
| Osteocalcin | 5’-TGAGTCCTGAGCAGCAG | 5’-TCTCTTCACTACCTCGCT |
| Osteopotin | 5’-CTGTTGCCTGTCTCTAAACC | 5’-CACCATCATCAAATTCTCCT |
| RUNX2 | 5’-GTCTCACTGCCTCTCACT | 5’-TACACACATCTCCTCCCTTC |
| Scleraxis | 5’-AGAAAGTTGAGCAAGGACC | 5’-CTGTCTGTACGTCCGTCT |
| GAPDH | 5’-GTATGACAACAGCCTCAAGAT | 5’-CCTTCCACGATACCAAAGTT |

|  |  | Flow cytometric analysis | | |
| --- | --- | --- | --- | --- |
| Clone | Isotype | A549 | TGFβ1-A549 | PBMC |
| E-Cadherin | ND | ++ | + (↓) | ND |
| N-cadherin | ND | + | + (↑) | ND |
| ER3-A7 | IgG1, κ | +++ | +++ (↑) | - |
| ER3-F3 | IgG2a, κ | + | + (↑) | - |
| ER4-D2 | IgG, κ | - | + (↑) | - |
| ER7-C3 | IgG, κ | + | + (↑) | - |
| ER7-A7 | IgG2a, κ | +++ | +++ (↑) | - |
| ER7-A8 | IgG1, κ | +++ | +++ (↑) | - |
| MR1-B1 | IgG2a, κ | ++ | ++ (↑) | - |
| MR1-G2 | IgG1, κ | + | ++ (↑) | - |
| MR11-B3 | IgG, κ | + | ++ (↑) | - |
| MR14-E5 | IgG2a, κ | + | ++ (↑) | - |
| MR14-F5 | IgG2a, κ | + | ++ (↑) | - |
| MR16-E3 | IgG2a, κ | ++ | ++ (↑) | - |
| MR17-B8 | IgG, κ | - | + (↑) | - |

**Table S2. Hybridoma clones specific to EMT-phenotypic A549 cells**

**Table S3.** **Expression profiles of MAb antigens on mesenchymal lineage cells with osteogenic potential**

|  | Flow cytometric analysis | | | |
| --- | --- | --- | --- | --- |
| Clone | hMSC | hFOB | U2OS | SAOS-2 |
| CD146 | ++ | +++ | +++ | +++ |
| ER3-A7 | + | ++ | ++ | ++ |
| ER3-F3 | + | ++ | ++ | ++ |
| ER4-D2 | - | - | - | - |
| ER7-C3 | - | - | + | - |
| ER7-A7 | + | ++ | ++ | ++ |
| ER7-A8 | +++ | +++ | +++ | +++ |
| MR1-B1 | + | + | ++ | + |
| MR1-G2 | ++ | - | + | - |
| MR11-B3 | - | - | - | - |
| MR14-E5 | ++ | + | + | - |
| MR14-F5 | + | + | ++ | ++ |
| MR16-E3 | ++ | +++ | + | + |
| MR17-B8 | - | - | - | - |

+ weak binding, ++ medium binding, +++ strong binding, − no binding

**Figure S1**

**
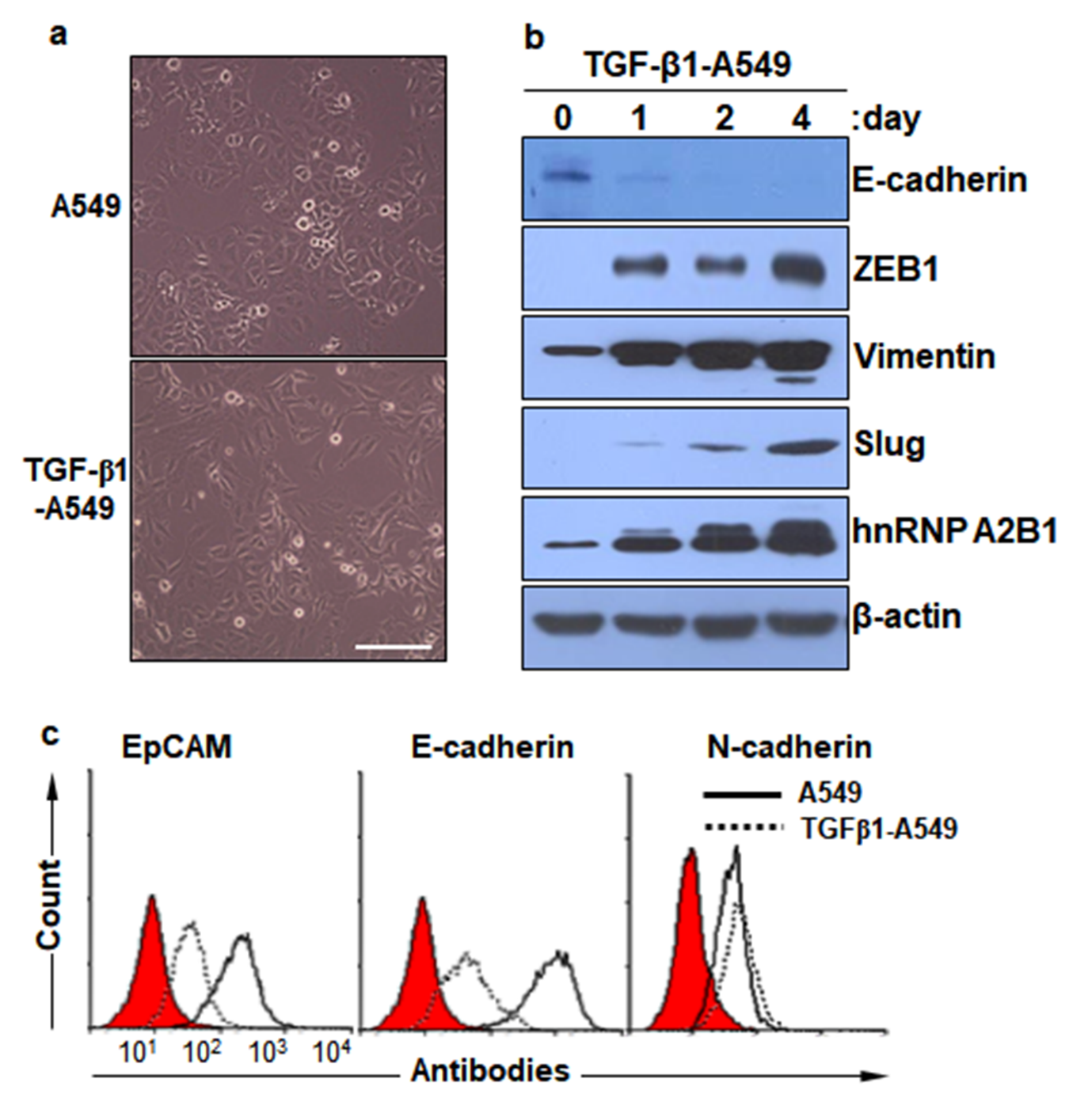
**

**Figure S1.** TGF-β1-treated A549 cells exhibit EMT phenotype. (**a**) Morphology of A549 and TGF-β1-treated A549 cells. A549 cells were treated with 5 ng/ml TGF-β1 for 4 days. The scale bar is 200 μm. (**b**) Expression of EMT markers in TGF-β1-treated A549 cells. TGF-β1-treated A549 cells were analyzed by Western blotting with the indicated antibodies. (**c**) Cell surface expression of EMT markers on TGF-β1-treated A549 cells. TGF-β1-treated A549 cells were analyzed by flow cytometry with the indicated antibodies. Red-filled histograms represent the isotype controls.

**Figure S2**


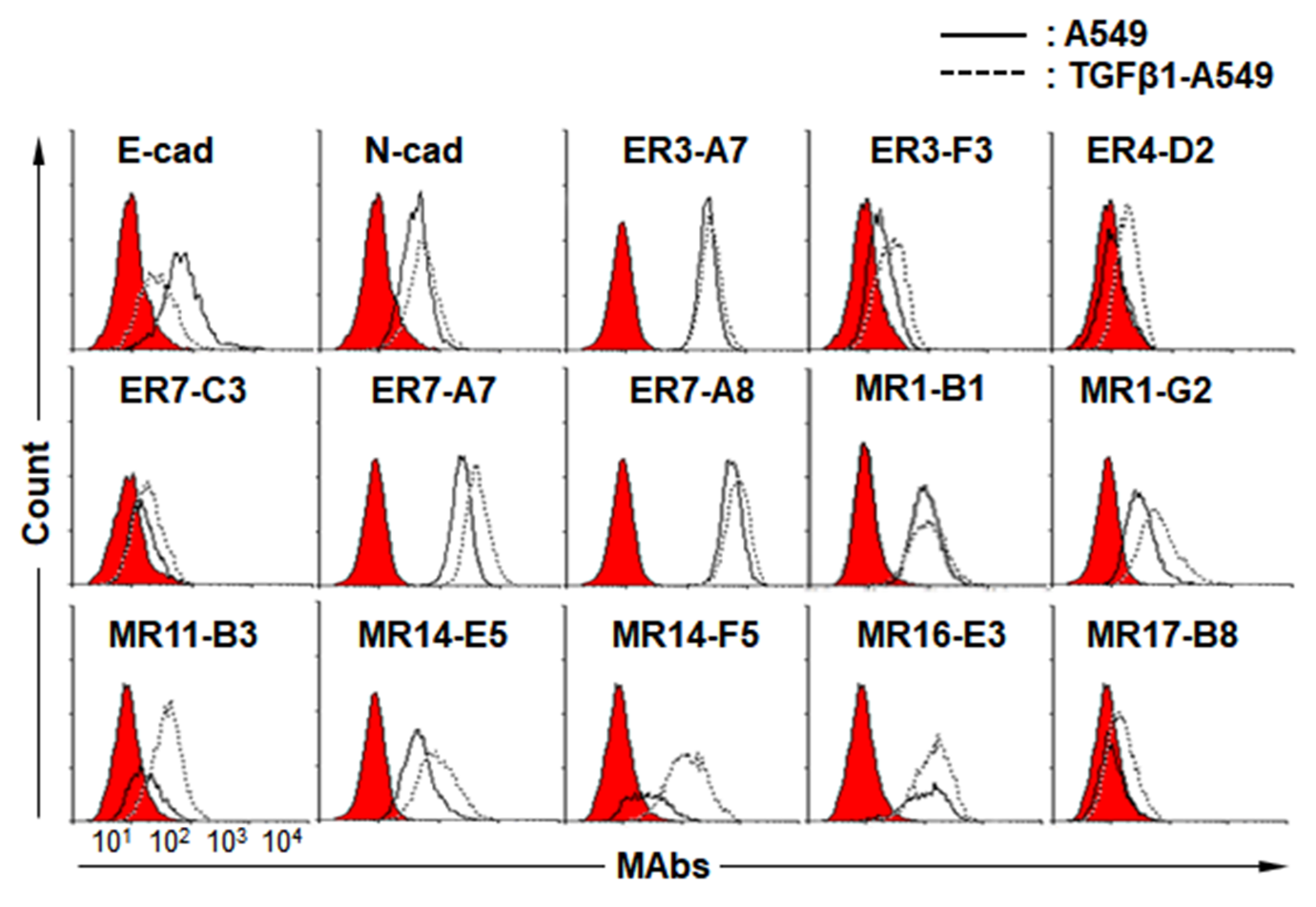


**Figure S2.** Screening of hybridoma clones specific to EMT-phenotypic A549 cells. Expression of MAb antigens was analyzed in A549 and TGF-β1-treated A549 cells by flow cytometry with the indicated MAbs. The expression of E-cadherin (E-cad) and N-cadherin (N-cad) was used as controls. Red-filled histograms represent the isotype controls.

**Figure S3**


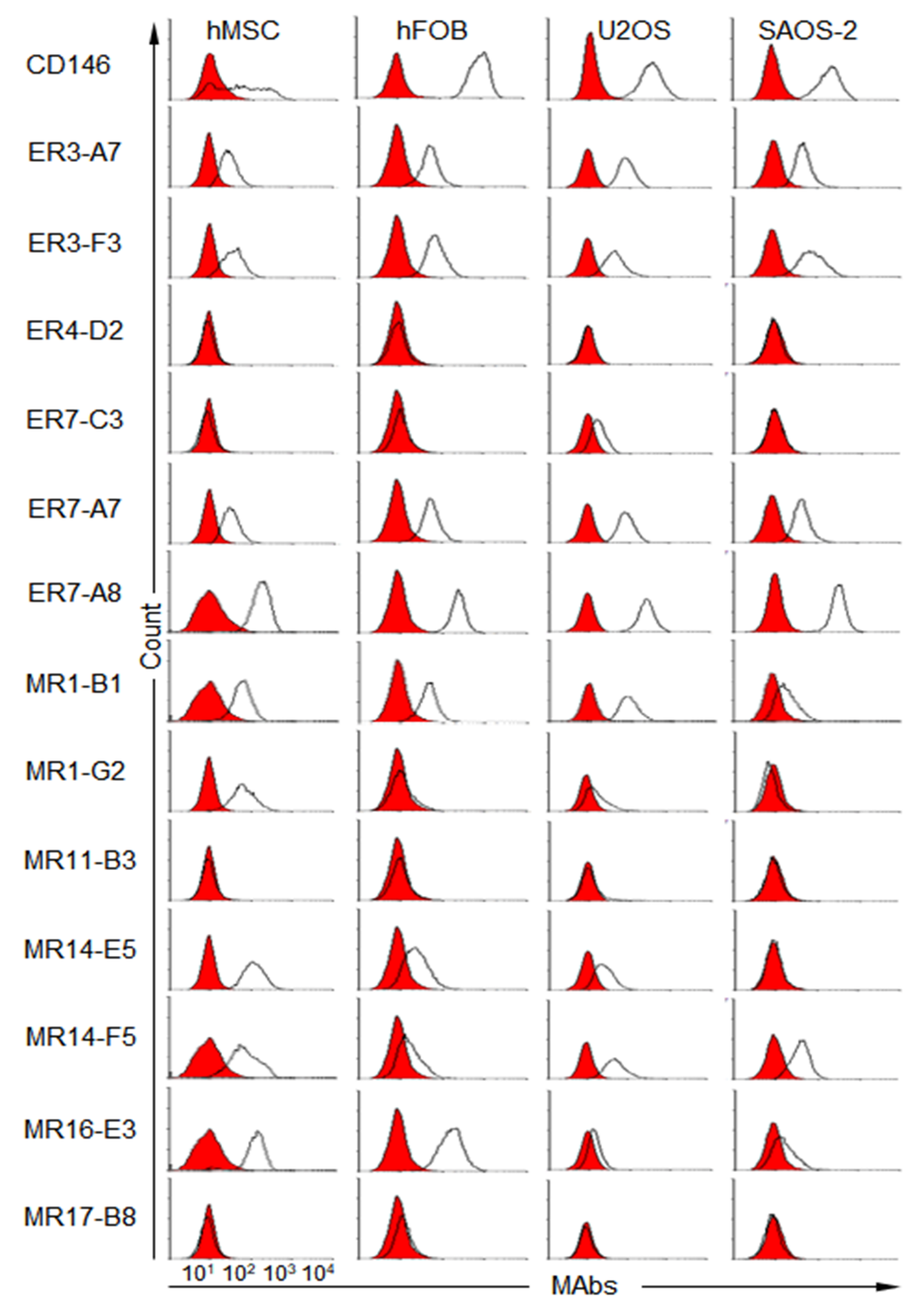


**Figure S3.** Cell surface expression of target antigens of selected MAbs in mesenchymal stem and progenitor cells with osteogenic potential. Cell surface expression of target antigens of selected MAbs was examined in two human osteogenic progenitor cells (hMSC and hFOB) and two human osteoblastic cancer cell lines (U2OS and SAOS-2) by flow cytometry with the indicated MAbs. Red-filled histograms represent the isotype controls.

**Figure S4**

**
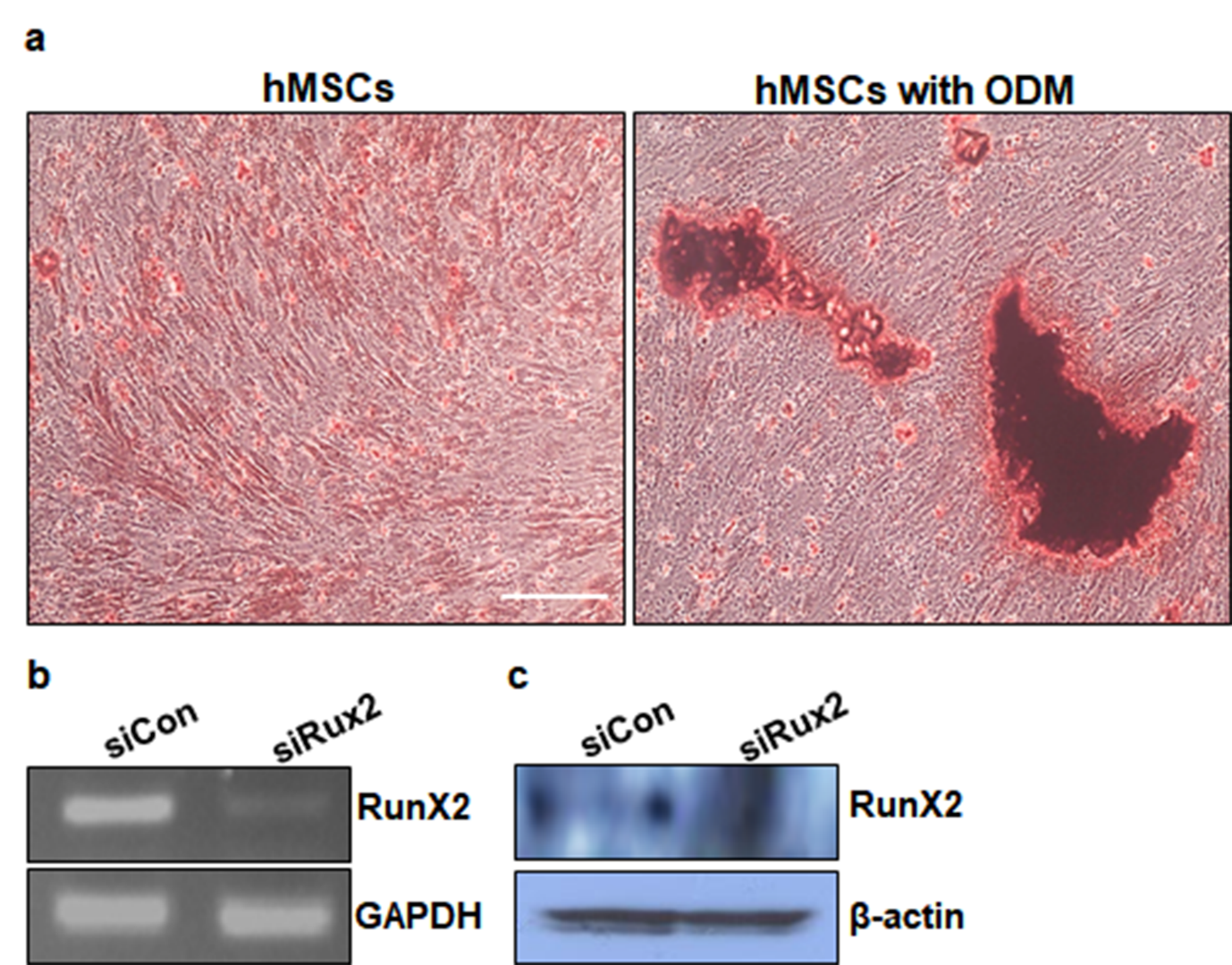
**

**Figure S4.** Alizarin Red S staining assay and knockdown efficiency of Runx2 in U2OS cells. (**a**) Alizarin Red S staining of hMSCs stimulated with ODM. hMSCs were incubated for 12 days with ODM, and calcium deposition and bone nodule were visualized as red color after the cells were stained with Alizarin Red S. The scale bar is 200 μm. (**b**) Knockdown efficiency of Runx2 in U2OS cells. After transfection of control siRNA or Runx2 siRNA, the expression of Runx2 gene was evaluated by RT-PCR (left panels) and by Western blotting (right panels). GAPDH or β-actin was used as a loading control.

**Figure S5**

**
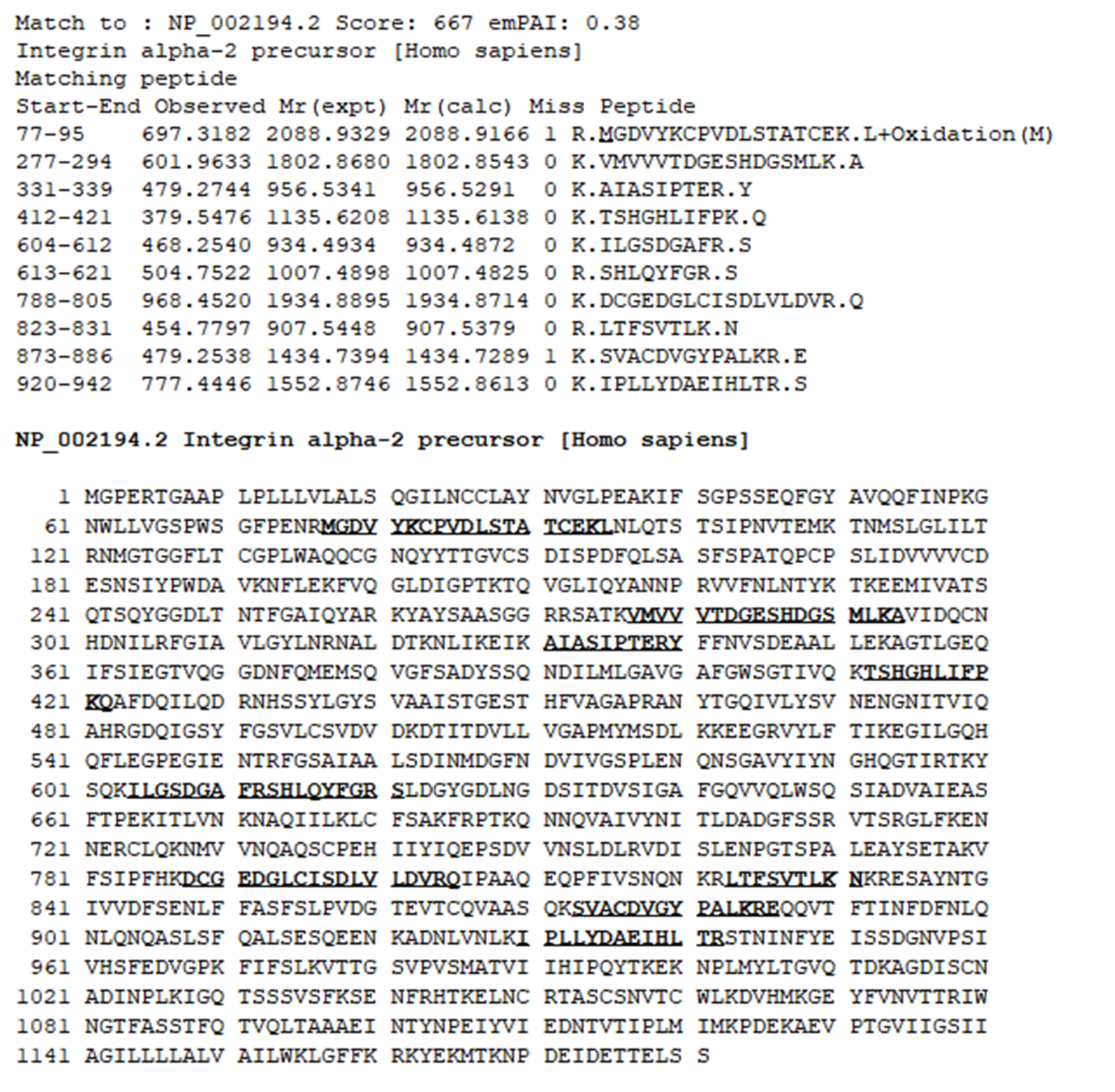
**

**Figure S5.** Mass spectrometric identification of MR14-E5 antigen after immunoprecipitation with ME14-E5. The approximately 150-kDa band from A549 cell lysates was treated with trypsin, and the resulting peptides were analyzed by mass spectrometry. Ten tryptic peptides (underlined) originating from the 150-kDa protein matched the integrin α2 precursor.

**Figure S6**


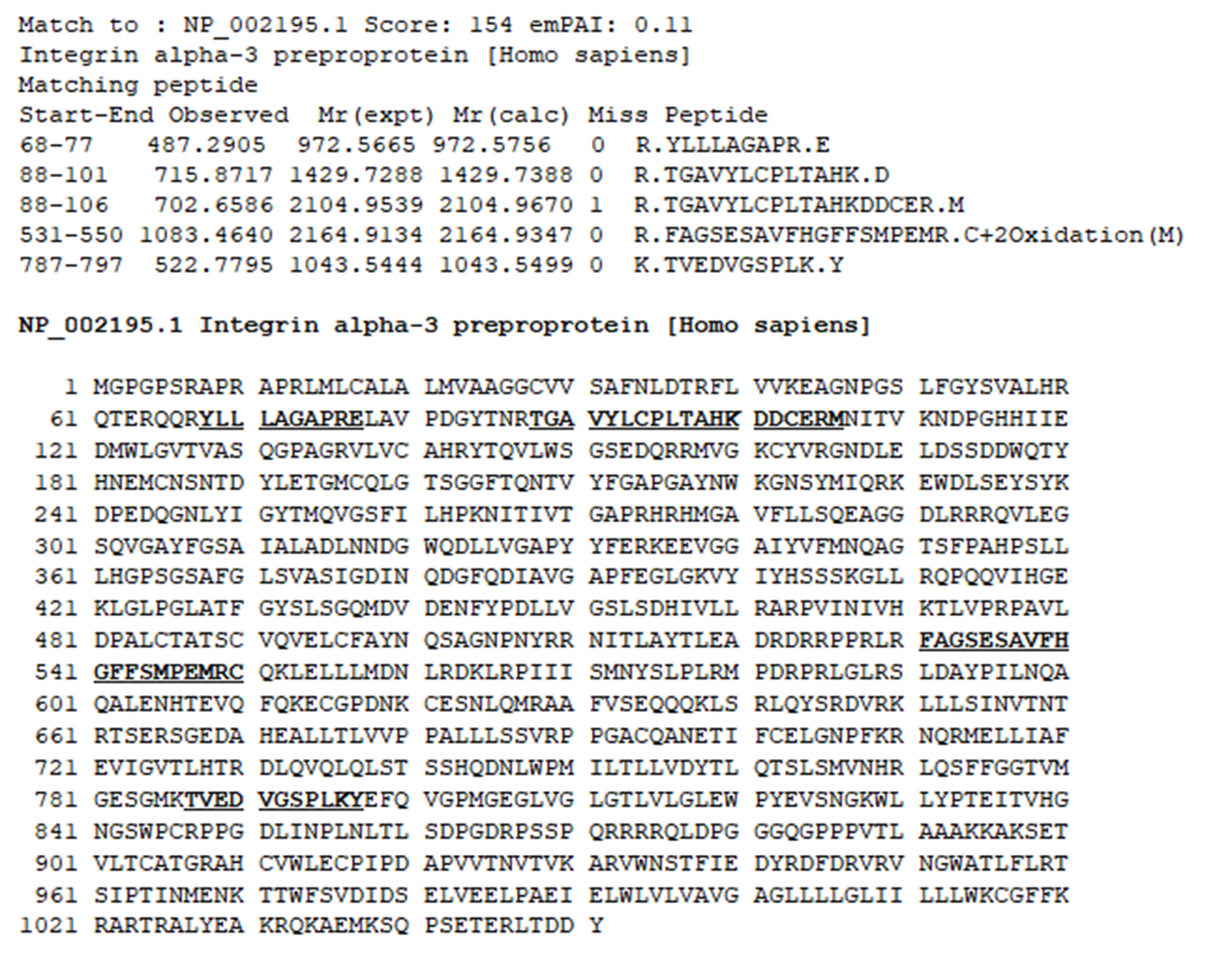


**Figure S6.** Mass spectrometric identification of ER7-A7 and ER7-A8 antigen after immunoprecipitation with ER7-A8. The approximately 130-kDa band from A549 cell lysates was treated with trypsin, and the resulting peptides were analyzed by mass spectrometry. Five tryptic peptides (underlined) originating from the 130-kDa protein matched the integrin α3 preproprotein.

**Figure S7**


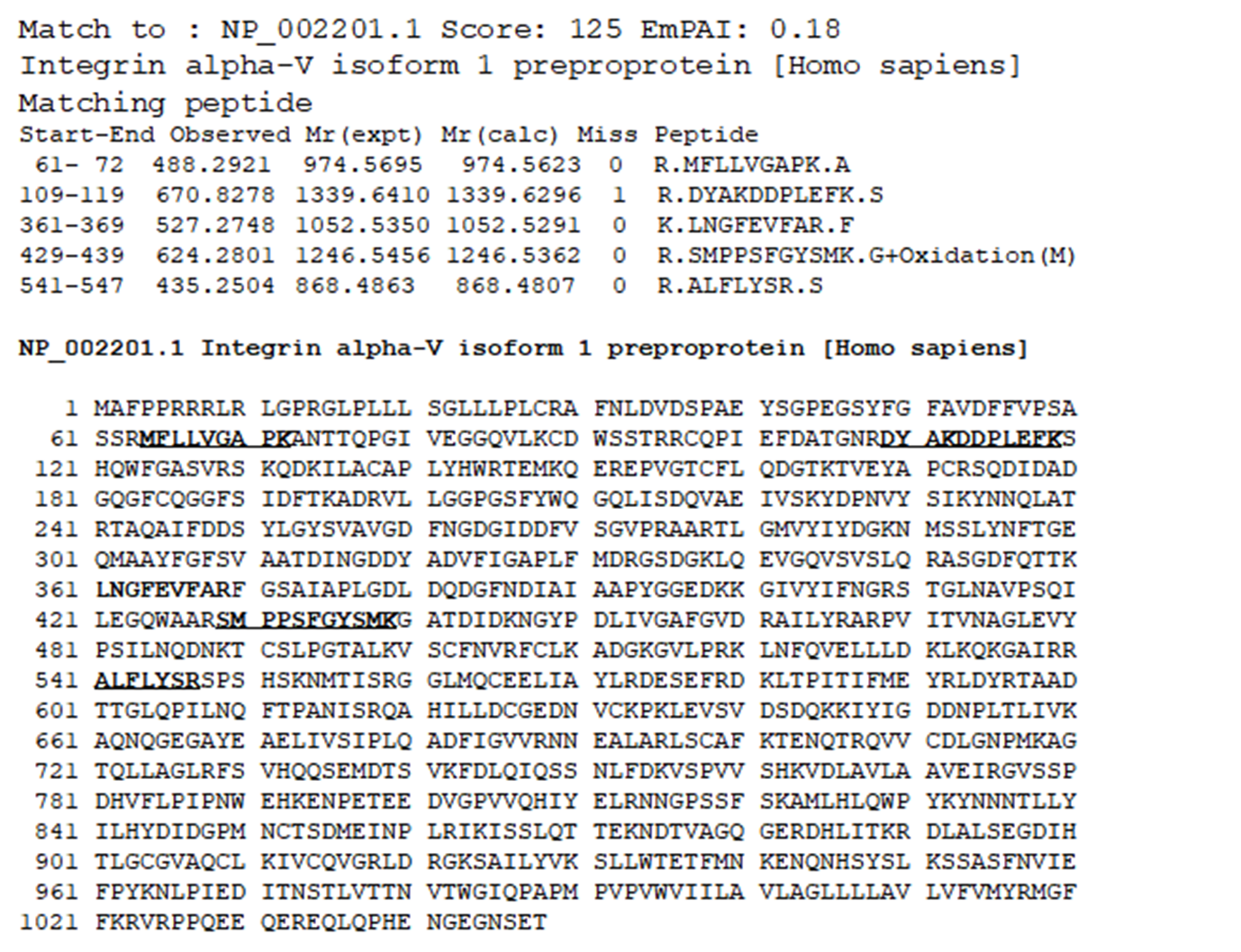


**Figure S7.** Mass spectrometric identification of MR1-B1 antigen after immunoprecipitation with MR1-B1. The approximately 130-kDa band from A549 cell lysates was treated with trypsin, and the resulting peptides were analyzed by mass spectrometry. Five tryptic peptides (underlined) originating from the 130-kDa protein matched the integrin αV isoform 1 preproprotein.

**Figure S8**

**
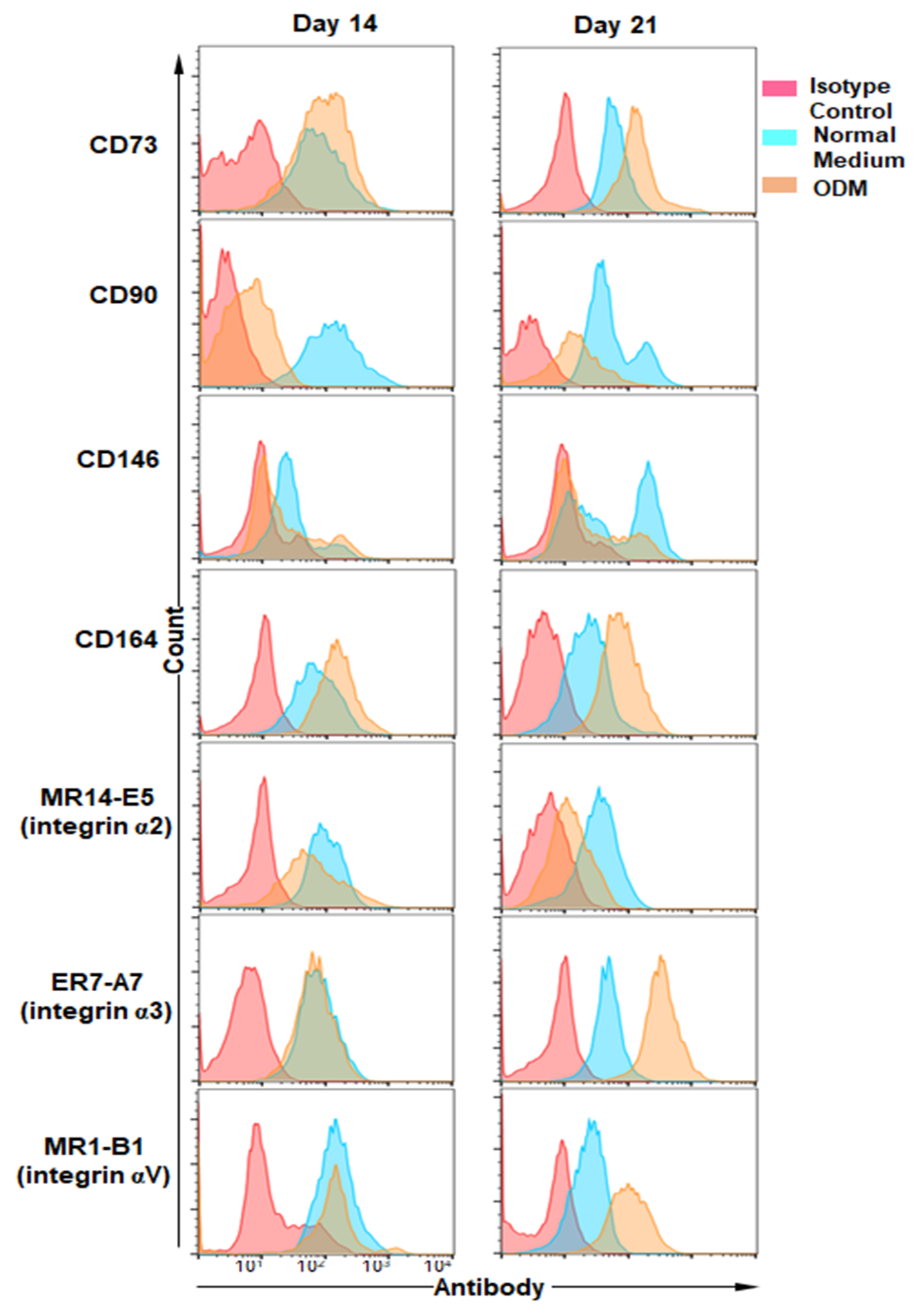
**

**Figure S8.** Expression changes of integrins and hMSC/OB surface markers upon osteogenic differentiation of hMSCs. hMSCs were incubated for 14, 21 days with ODM, and SB431542 was added to ODM after 7 days of the osteogenic differentiation. Integrins (α2, α3, αV), hMSC/OB surface markers (CD73, CD90, CD146 and CD164) were analyzed in undifferentiated (normal growth medium) and differentiated hMSCs (ODM) by flow cytometry. Red-filled histograms represent isotype controls.

**Figure S9**


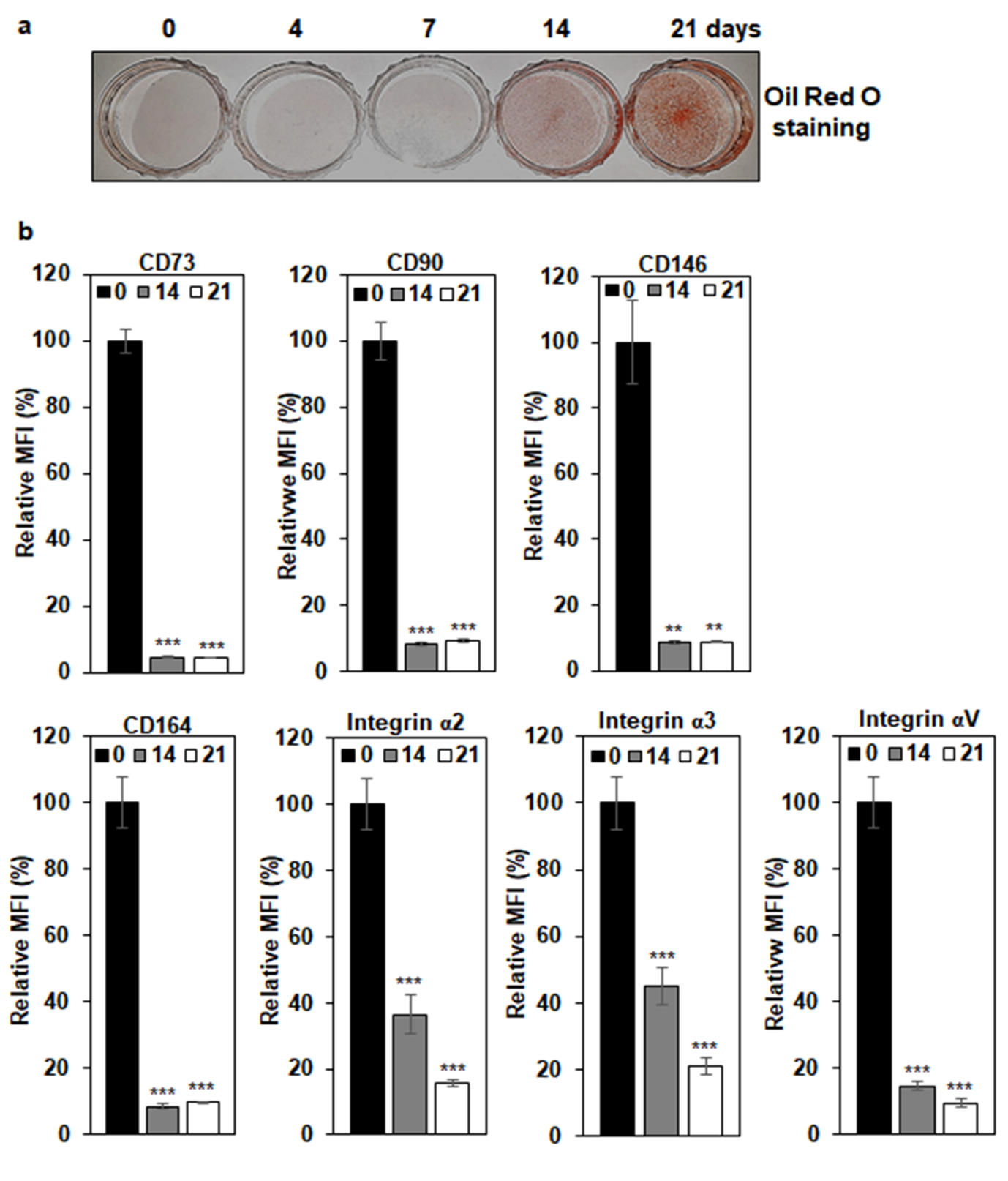


**Figure S9**. Expression changes of integrin αV, α2, α3 and osteogenic markers during adipogenic differentiation of hMSCs. (**a**) Oil Red O staining of adipocytes in differentiated hMSCs. hMSCs were incubated for 21 days with ADM. Lipid content was visualized as red color after the cells were stained with Oil Red O. (**b**) Expression changes of integrins and hMSC/OB surface markers during adipogenic differentiation of hMSCs. Integrins (MR14-E5, ER7-A7, MR1-B1) and hMSC/OB surface markers (CD73, CD90, CD146 and CD164) were analyzed in differentiated hMSCs by flow cytometry. Values are depicted as a relative MFI of differentiated hMSCs at the indicated days compared to hMSCs cultured in normal medium. **, p < 0.01; ***, p < 0.005.

**Figure S10**


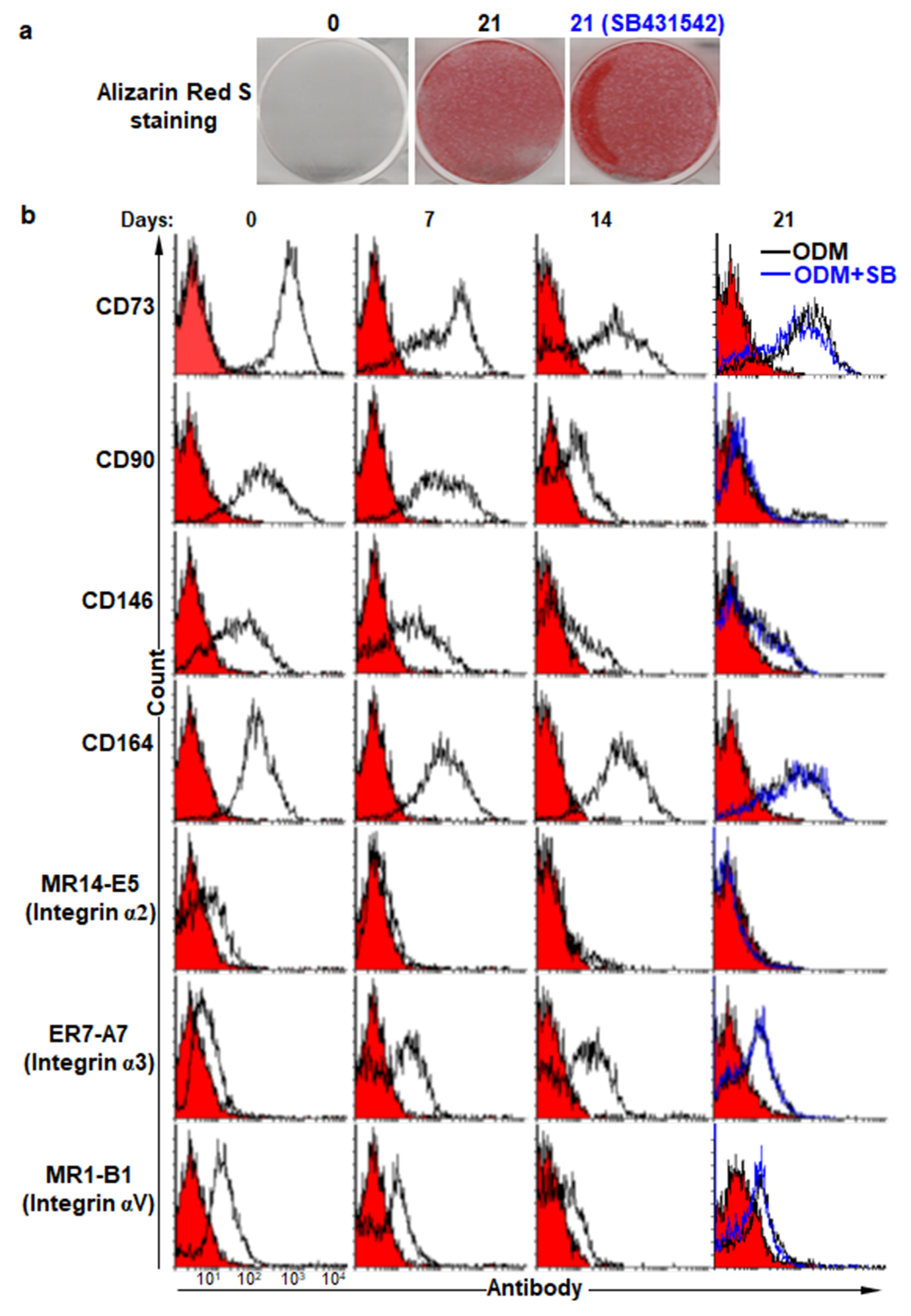


**Figure S10**. Expression changes of integrins and hMSC/OB surface markers upon osteogenic differentiation of hMSCs in the absence and presence of SB431542. (a) Alizarin Red S staining of hMSCs stimulated with ODM. hMSCs were incubated for 21 days with ODM in the absence and presence of SB431542. SB431542 was included in ODM after 14 days of osteogenic differentiation and calcium deposition and bone nodule were visualized as red color after the cells were stained with Alizarin Red S. (b) Flow cytometric analysis of hMSCs cultured in ODM in the absence and presence of SB431542. hMSCs were incubated for 7, 14, 21 days with ODM, and SB431542 was added to ODM after 14 days of osteogenic differentiation. Integrins (α2, α3, αV), hMSC/OB surface markers (CD73, CD90, CD146 and CD164) were analyzed by flow cytometry. Red-filled histograms represent isotype controls.
